# Specific detection of fission yeast primary septum reveals septum and cleavage furrow ingression during early anaphase independent of mitosis completion

**DOI:** 10.1371/journal.pgen.1007388

**Published:** 2018-05-29

**Authors:** Juan Carlos G. Cortés, Mariona Ramos, Mami Konomi, Iris Barragán, M. Belén Moreno, María Alcaide-Gavilán, Sergio Moreno, Masako Osumi, Pilar Pérez, Juan Carlos Ribas

**Affiliations:** 1 Instituto de Biología Funcional y Genómica and Departamento de Microbiología y Genética, Consejo Superior de Investigaciones Científicas (CSIC) / Universidad de Salamanca, Salamanca, Spain; 2 Laboratory of Electron Microscopy/Bio-imaging Centre, and Department of Chemical and Biological Sciences, Japan Women's University, Mejirodai, Bunkyo-ku, Tokyo, Japan; 3 Centro Andaluz de Biología del Desarrollo, Universidad Pablo de Olavide/Consejo Superior de Investigaciones Científicas, Sevilla, Spain; 4 NPO: Integrated Imaging Research Support, Hirakawa-cho, Chiyoda-ku, Tokyo, Japan; Univ. of Mass Medical School, UNITED STATES

## Abstract

It is widely accepted in eukaryotes that the cleavage furrow only initiates after mitosis completion. In fission yeast, cytokinesis requires the synthesis of a septum tightly coupled to cleavage furrow ingression. The current cytokinesis model establishes that simultaneous septation and furrow ingression only initiate after spindle breakage and mitosis exit. Thus, this model considers that although Cdk1 is inactivated at early-anaphase, septation onset requires the long elapsed time until mitosis completion and full activation of the Hippo-like SIN pathway. Here, we studied the precise timing of septation onset regarding mitosis by exploiting both the septum-specific detection with the fluorochrome calcofluor and the high-resolution electron microscopy during anaphase and telophase. Contrarily to the existing model, we found that both septum and cleavage furrow start to ingress at early anaphase B, long before spindle breakage, with a slow ingression rate during anaphase B, and greatly increasing after telophase onset. This shows that mitosis and cleavage furrow ingression are not concatenated but simultaneous events in fission yeast. We found that the timing of septation during early anaphase correlates with the cell size and is regulated by the corresponding levels of SIN Etd1 and Rho1. Cdk1 inactivation was directly required for timely septation in early anaphase. Strikingly the reduced SIN activity present after Cdk1 loss was enough to trigger septation by immediately inducing the medial recruitment of the SIN kinase complex Sid2-Mob1. On the other hand, septation onset did not depend on the SIN asymmetry establishment, which is considered a hallmark for SIN activation. These results recalibrate the timing of key cytokinetic events in fission yeast; and unveil a size-dependent control mechanism that synchronizes simultaneous nuclei separation with septum and cleavage furrow ingression to safeguard the proper chromosome segregation during cell division.

## Author summary

Fission yeast cytokinesis requires the invagination of the equatorial plasma membrane (cleavage furrow ingression) coupled to the synthesis of a special wall structure named septum (septation). Despite Cdk1 kinase is inactivated in early anaphase, it is believed that cleavage furrow ingression and septation onset require anaphase progression and mitosis completion, only initiating after the complete activation of the Hippo-like septation initiation network (SIN) after telophase onset. Here, we studied the precise timing of septation start with respect to mitosis through specific septum-staining and electron microscopy. We found that septum and cleavage furrow ingression initiate in early anaphase, showing first a slow ingression rate during anaphase B, and increasing to a fast ingression rate after telophase onset. Thus, mitosis and cleavage furrow ingression are not concatenated but simultaneous events in fission yeast. The timing of septation correlated with cell size and depended on the level of cytoplasmic activators like SIN Etd1 and Rho1. We further analyzed the mitotic mechanisms that control the septation onset during early anaphase. Cdk1 directly regulated the timing of septation onset during early anaphase, and the low SIN activity present after Cdk1 inactivation was enough to trigger septation. Globally, these results recalibrate the timing of the main cytokinetic events of fission yeast and reveal a size-dependent control mechanism that synchronizes simultaneous nuclei separation with septum and cleavage furrow ingression.

## Introduction

The division of a cell into two genetically identical daughter cells requires the accurate coordination of events such as the entry into mitosis, chromosome segregation, and cytokinesis. Thus, precise timing of the late mitotic events is critical for faithful chromosome segregation and genome integrity. The events occurring during mitosis exit follow a strictly defined order. First, chromosome arms reach a state of maximum condensation as the spindle extends to pull chromosomes apart. Next, at the end of anaphase B the chromosomes decondense, the spindle breaks down, and cleavage furrow ingression begins [1–3]. Progression through the cell cycle is controlled by fluctuations in the activity of cyclin-dependent kinase (Cdk) complexes, which are dependent upon periodic expression of cyclin subunits. At anaphase B onset, cyclin is degraded by the anaphase-promoting complex which allows anaphase B progression and the exit from mitosis [4]. Cdk1 is considered a global inhibitor of cytokinesis, which depends on the conclusion of the preceding mitosis [5, 6]. Thus, the expression of a non-degradable cyclin B in fission yeast blocks the cells in anaphase B and abolishes cytokinesis [7].

In fungi the spindle pole bodies (SPBs) are functionally analogous to centrosomes. The septation initiation network (SIN) signals from the cytoplasmic face of the SPB to activate both septum and cleavage furrow ingression after telophase onset [8]. SIN equivalents are conserved in the yeast MEN and metazoans Hippo pathways [8, 9]. SIN signaling requires activation of the GTPase Spg1 [10]. Byr4 and Cdc16 form a two-component GTPase-activating protein complex, which restrains septation in interphase by keeping Spg1 in the inactive GDP-bound state [11, 12]. Upon entry into mitosis, phosphorylation of Byr4 by Cdk1 facilitate Byr4-Cdc16 removal from the SPBs [13], allowing Spg1 to become active at both SPBs [12, 14]. Then active Spg1-GTP recruits Cdc7 kinase to both SPBs in early mitosis [14]. However, further activation of the SIN during mitosis is blocked by Cdk1 activity. At anaphase B onset, the loss of Cdk1 activity allows the increase of SIN activity [15], being Spg1 inactivated by Byr4-Cdc16 at one of the two SPBs, and Cdc7 disappearing from that inactive SPB [12, 16]. The levels of Cdc7 in the active SPB gradually increase and peak at the end of anaphase B, in a process that depends on the SIN Etd1 [17, 18]. The fact that Etd1 localizes to the cortex and cytoplasm but not to the SPB led to propose that after the spindle is fully elongated, the release of Etd1 from the pole cortex completes SIN activation probably by binding to cytoplasmic Spg1 [18, 19]. Maximal SIN activation at the end of anaphase B causes the relocation of NDR-family kinase Sid2 from the SPB to the division site, where it presumably triggers septum synthesis and furrow ingression by activating the conserved GTPase Rho1 [9, 20, 21].

Cytokinesis in fungi requires the membrane invagination combined with the closure of a conserved actomyosin ring (AR) and the synthesis of a special wall structure named division septum [22, 23]. Fission yeast cytokinesis is divided into four consecutive steps: 1) an equatorial band of nodes is established before entry into mitosis; 2) the nodes condense into a compact AR in anaphase A; 3) the compacted AR maturates acquiring new proteins during anaphase B; and 4) septation begins and the AR starts to close after the fully elongated spindle breaks down [24, 25]. The septum is mainly composed of polysaccharide chains of α- and β-glucans, displaying a three-layered structure with a central disk called the primary septum (PS) and flanked by the secondary septum on each side [23]. The septum is built by at least three essential glucan synthases (GSs). Bgs1/Cps1 is responsible for the linear-β(1,3)glucan (L-BG) synthesis of the PS [26], and Bgs4 and Ags1/Mok1 cooperate to form the secondary septum. In addition, Bgs4 couples AR closure with PS ingression, while Ags1 confers to the PS the mechanical strength required for a gradual and safe cell separation [27, 28].

L-BG is exclusively detected in the PS structure, where this polysaccharide specifically interacts with and has high affinity to the fluorochrome calcofluor white (CW) [26]. Therefore, CW has shown to be a valuable tool to precisely visualize cytokinesis both temporally and spatially [27, 28]. In this study, CW was used to examine the timing of septum synthesis start with respect to mitosis in fission yeast. Contrary to the general belief that septum synthesis initiates after telophase onset, we have determined by fluorescence and electron microscopy that both septum and cleavage furrow start to ingress at early anaphase B, occurring long before the chromosome masses reach the cell tips and the spindle breaks down at the start of telophase. In addition, we describe that septation presents two distinct ingression rates: very slow during anaphase B and much faster after telophase onset. We also found that the timing of septation initiation with regard to mitosis scales with the cell size in an Etd1 and Rho1 dependent manner, and depends on Cdk1 inactivation, which allows the SIN activation and septation onset during early anaphase B. Taken together; our findings reveal the existence of a size-dependent control mechanism that helps to coordinate simultaneous chromosome mass separation with septum and cleavage furrow ingression, recalibrating the timing and regulation of crucial events of fission yeast cytokinesis.

## Results

### The initiation of septum and cleavage furrow ingression overlaps with the early stages of anaphase B in fission yeast

**Fig 1:**
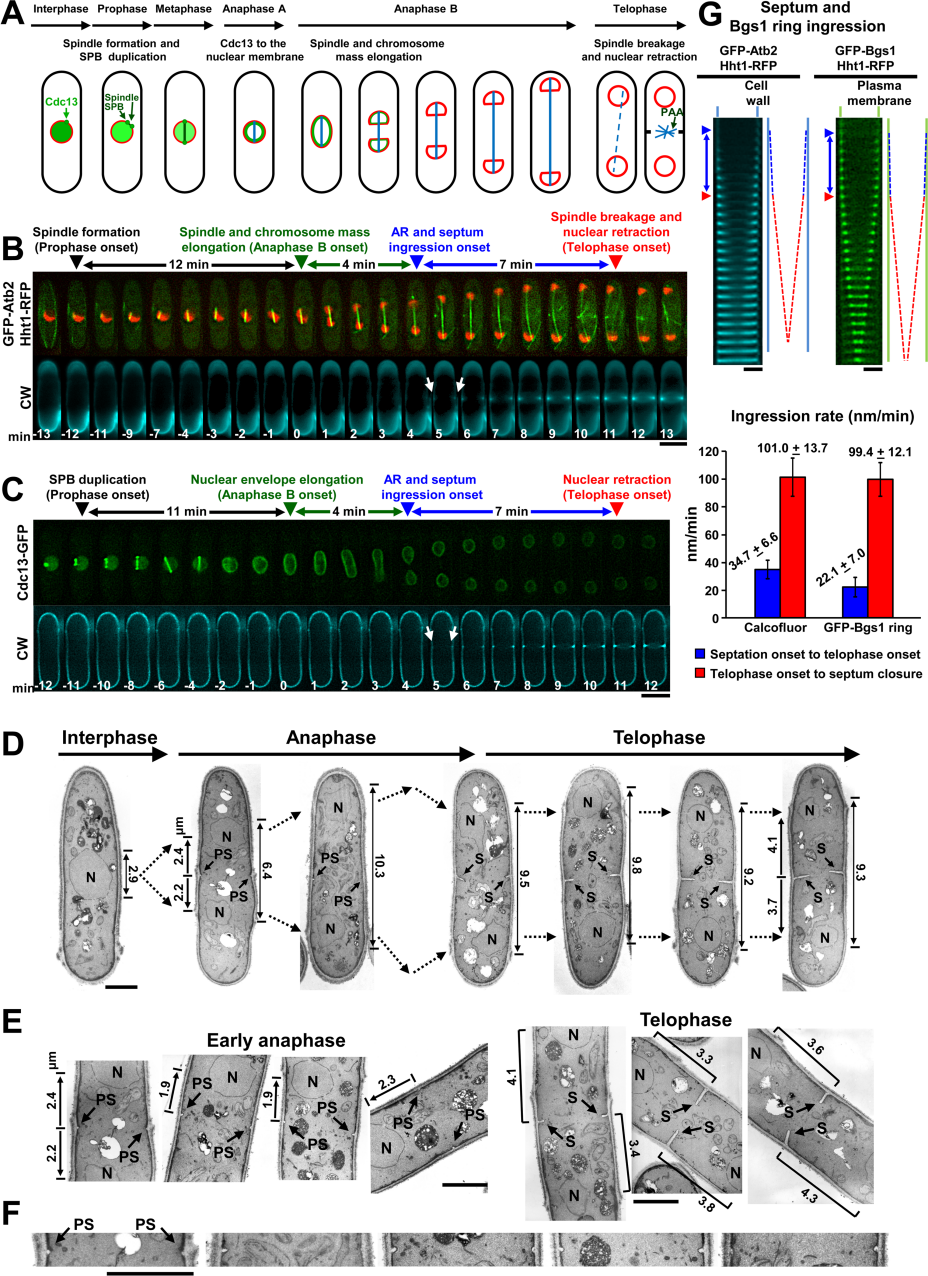
Septum and cleavage furrow initiation overlaps with the early stages of anaphase B in fission yeast. **(A)** Scheme of the mitotic phases analyzed in this study. Prophase begins with the duplication of the spindle pole body (SPB) and continues with the elongation of the mitotic spindle (blue). In metaphase the spindle does not change in length. The cyclin B Cdc13 (green) localizes to the spindle pole bodies (SPB) and mitotic spindle during prophase and metaphase. In anaphase A, Cdc13 relocates to the nuclear envelope (red), and is then degraded. Anaphase B starts with spindle and chromosome mass elongation. Finally, spindle disassembly and retraction of the nuclei from the poles indicate the exit from mitosis and the start of telophase. During telophase the nuclei are stably located away from the cell middle by the postanaphase array (PAA) of microtubules (MT). **(B, C)** Septum deposition starts at early anaphase B, long before the spindle disassembly. Wild-type cells carrying Hht1-RFP (histone H3) and GFP-Atb2 (MT) or Cdc13-GFP were grown in YES with Calcofluor white (CW, 5 μg ml^-1^) at 28°C and imaged by time-lapse fluorescence microscopy (1 medial z slice, 1 min elapsed time). The data of this figure are developed in Table 1, Table 3 and S1 Table. White arrow: first CW-stained septum synthesis. Arrowheads: black, prophase onset; green, anaphase B onset (time 0); blue, septum deposition onset (time immediately before septum detection with CW); red, telophase onset. **(D-F)** Septum formation during early anaphase B implies concomitant plasma membrane invagination (cleavage furrow formation) and closure of a contractile actomyosin ring (AR). Transmission electron microscopy images of wild-type cells as in B (D) and magnifications of the division site (E, F), showing the initial primary septum (PS) emergence in anaphase B and the septum (S) in telophase, were examined. A higher magnification of the PS of early anaphase B cells of D and E is shown (F). The distance between nucleus middle (N) and septum is shorter in cells with a nascent PS (arrows to the left, 1.9 to 2.4 μm, early anaphase B, nuclei close to the cell middle) than in cells with larger septum (brackets, 3.3 to 4.3 μm, telophase, nuclei stably located away from the cell middle). The spindle size, calculated as the distance between nuclei ends (arrows to the right), is shown. **(G)** The septum exhibits two different ingression rates, very slow during anaphase B and much faster during telophase. Top, kymographs and schemes showing the progression of septum and GFP-Bgs1 ring ingression during both anaphase B and telophase. Wild-type cells carrying Hht1-RFP and GFP-Atb2 (left; n = 6, ≥25 cells) or GFP-Bgs1 (right; n = 3, ≥22 cells) were grown and imaged as in B. Arrowheads are as in B. Bottom, graph showing the rate of septum and GFP-Bgs1 ring ingression during the indicated mitotic intervals. Error bars indicate standard deviation (SD). Bars, 5 μm (B, C) and 2 μm (D-G).

Based on the visual detection of AR closure by standard fluorescence microscopy, it is considered that AR closure and septation initiate simultaneously after spindle disassembly at the onset of telophase [25]. Here the onset of septum synthesis in fission yeast has been investigated by visualizing the emergence of the PS fluorescence through specific CW staining with respect to all possible mitotic events. Table 1, Table 2 and S1 Table encapsulate the complete time intervals between septum synthesis initiation and the mitotic events defined in Fig 1A of the main strains and growth conditions analyzed in this study. To precisely detect possible alterations in the time of septation onset (blue arrowhead indicates the time immediately before the first PS detection, Fig 1B and 1C), and to avoid the variations that might occur in the timing with respect to distant mitotic events, the anaphase B onset was considered as time zero (green arrowhead, Fig 1B and 1C). This event was the closest and most accurate for measuring the elapsed time until septation onset. When required, other events were considered as time zero as specified in text and figures.

**Table 1:** Time intervals of the different mitotic and cytokinetic events at 28°C.

Strain	Prophase^1^	Metaphase/Anaphase A^1^	Anaphase B^1^	
			Septation onset^2^	Spindle disassembly^2^
**Wild-type** (n = 18, 126 cells)^3^	6.5 ± 1.2	5.9 ± 0.5	5.7 ± 1.0 *(18*.*1)*^4^	7.1 ± 1.1 *(25*.*2)*^4^
***cdc13***^***+***^***-GFP*** (n = 3, 20 cells)	5.5 ± 0.5	6.2 ± 0.4	5.7 ± 0.8^NS^ *(17*.*4)*	5.2 ± 1.2 *(22*.*6)*
***cdc15-GFP*** (n = 4, 14 cells)	6.3 ± 0.9	6.3 ± 1.5	11.2 ± 1.4*** (23.8)	3.4 ± 1.3 *(27*.*2)*
***sid2-250*** (n = 2, 30 cells)	n.d.	n.d.	9.1 ± 1.1*** (n.d.)	3.5 ± 2.3 *(n*.*d*.*)*
***myp2*Δ** (n = 2, 10 cells)	6.5 ± 0.5	6.8 ± 1.7	7.4 ± 0.8*** (20.7)	6.1 ± 1.6 *(26*.*8)*

Values are minutes ± SD.

^1^ Mitotic phases are as described in Fig 1A.

^2 ^Elapsed time between septation onset and steps of anaphase B.

^3 ^The value n is the number of independent experiments performed in different days in each case.

^4^ The values underlined in parenthesis are the total minutes since the mitotic entry in prophase.

Asterisks indicate significant statistical difference in the septation onset of the corresponding strain compared to wild-type by the Student´s test:

* p <0.05

** p <0.01

*** p <0.001; NS: not significant difference (p >0.05).

n.d., not determined.

**Table 2:** Time intervals of the different mitotic and cytokinetic events at 25°C.

Strain	Prophase^1^	Metaphase/Anaphase A^1^	Anaphase B^1^	
			Septation onset^2^	Spindle disassembly^2^
**Wild-type** (n = 6, 51 cells)^3^	6.6 ± 0.8	8.4 ± 1.3	7.8 ± 1.5 *(**22*.*8**)*^4^	8.7 ± 2.3 *(**31*.*5**)*^4^
***wee1-50*** (n = 3, 15 cells)	7.3 ± 1.6	6.6 ± 1.6	6.0 ± 1.4**(*19*.*9**)*	11.0 ± 2.5 *(**30*.*9**)*
***cdc2-3W*** (n = 3, 16 cells)	6.2 ± 1.0	7.7 ± 1.7	6.1 ± 1.6**(*20*.*0**)*	8.8 ± 1.8 *(**28*.*8**)*
***cdc10-119*** (n = 3, 11 cells)	11.1 ± 1.6	8.2 ± 2.7	9.2 ± 1.2**(*28*.*5**)*	17.5 ± 3.4 *(**46*.*0**)*
***cdc25-22*** (n = 6, 32 cells)	10.1 ± 1.1	10.1 ± 3.3	10.9 ± 2.0*** *(**31*.*1**)*	16.8 ± 1.6 *(**47*.*9**)*

Values are minutes ± SD.

^1^ Mitotic phases are as described in Fig 1A.

^2 ^Elapsed time between septation onset and steps of anaphase B.

^3^ The value n is the number of independent experiments performed in different days in each case.

^4^ The values underlined in parenthesis are the total minutes since the mitotic entry in prophase.

Asterisks indicate significant statistical difference in the septation onset of the corresponding strain compared to wild-type by the Student´s test:

* p <0.05

** p <0.01

*** p <0.001; NS: not significant difference (p >0.05).

The emergence of PS was examined in different wild-type backgrounds to ensure the obtained data was reproducible. Labeling of certain ring proteins can compromise the AR function [29, 30]. Thus, to avoid artifacts in the timing of septation onset, the analyzed strains did not contain any tagged ring protein. In all strains the PS was reproducibly detected in early anaphase B, close to the transition to mid-anaphase B (Fig 1B and 1C, S1 Fig, Table 1 and Table 2) and long before the spindle breakage and telophase onset (red arrowhead, Fig 1B and 1C).

It is assumed that initial septum deposition is tightly coupled with cleavage furrow ingression. Therefore, to determine if the small septa observed during early anaphase really grew inwards, electron microscopy analysis was performed (Fig 1D–1F). Cells in anaphase B and telophase, as shown by the close or distant nuclei position and by the presence and size of the septum, also unequivocally demonstrated that septum formation occurred in early anaphase B. Importantly, the presence of small PS from 32 to 137 nm in length showed that the nascent septa grow inwards. This indicates that membrane invagination and AR closure at the septum edge have also occurred simultaneously (Fig 1D–1F). In agreement, fluorescence microscopy showed that both CW-stained septum and GFP-Bgs1 ring presented a slow ingression rate during anaphase B, increasing about 3-fold after telophase onset (red arrowhead) and coinciding with the visual detection of AR closure (Fig 1G). Thus, the start of septum and cleavage furrow ingression overlaps with the early stages of chromosome mass separation during anaphase B, independent of mitosis conclusion.

### The timings of septation onset and septum closure correlate with the cell size

**Fig 2:**
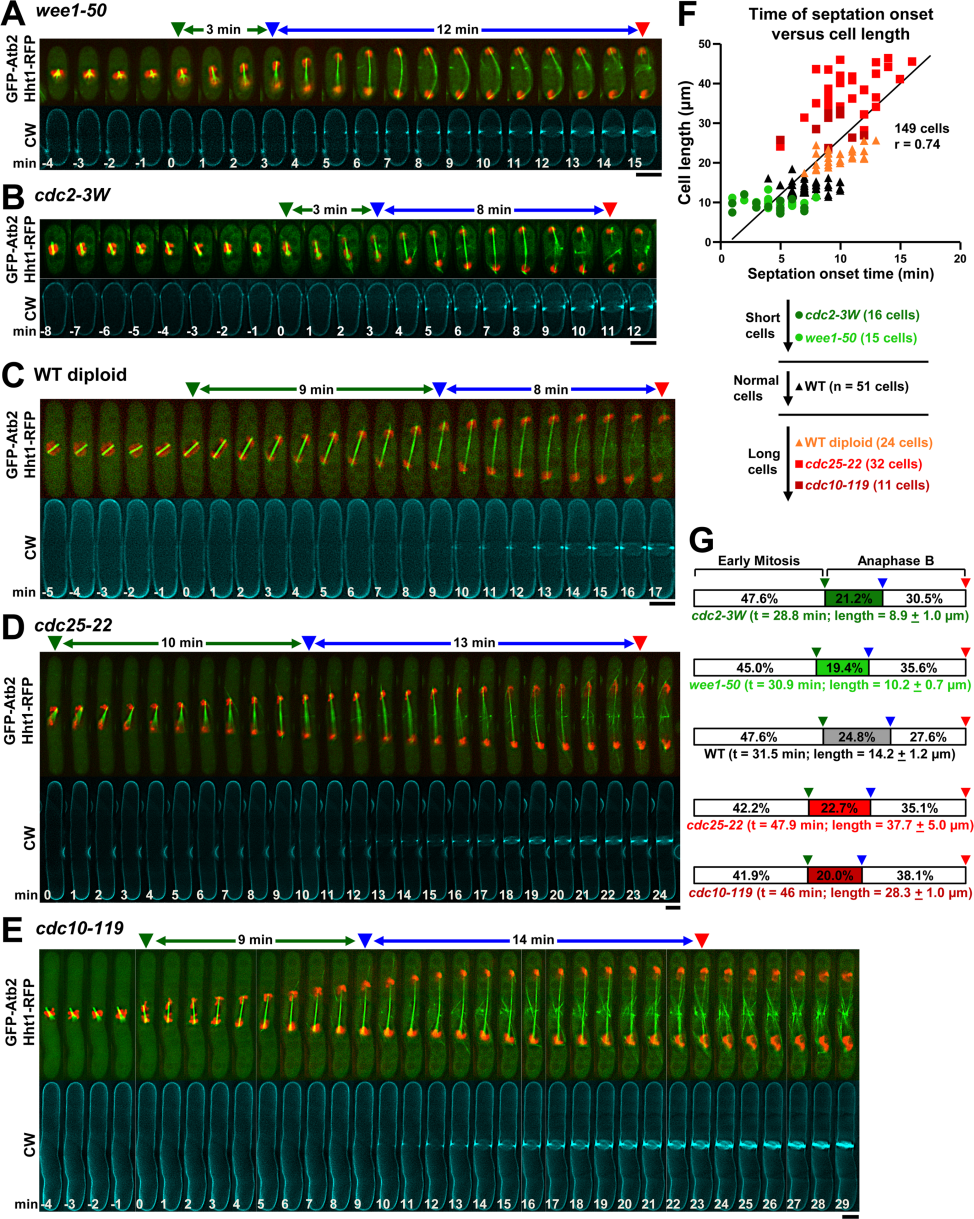
The start of septation scales with anaphase B progression and cell size in fission yeast. Cell cycle-short *wee1-50* **(A)** and *cdc2-3W* **(B)**, wild-type long diploid **(C)**, and cell cycle-long *cdc25-22* **(D)** and *cdc10-119* **(E)** cells were grown in YES at 25°C (A-C) or released at 25°C after 3.5 h of cell cycle arrest at 36°C (D, E), and imaged as in Fig 1. **(F)** The timing of septation correlates linearly with the cell length. The time of septum deposition initiation since anaphase B onset was plotted against the cell size in the indicated strains. The total cell number and Pearson product-moment correlation coefficient (r) are shown in the plot. The data of this figure are developed in Table 2, Table 3 and S1 Table. **(G)** The percentages of elapsed anaphase B at the time of septum synthesis onset with respect to the total time of mitosis are similar in wild-type, long and short cells. A scheme of the elapsed time from anaphase B to septation onset as percentage of total mitosis is shown (see also S1 Table). The elapsed time in mitosis (t, min) and cell length (μm ± SD) of each strain are shown. Symbols are as in Fig 1. Bars, 5 μm.

The newly described start of the septation process overlaps with the spindle elongation, and given that spindle length scales with cell size [31], the correlation between the timing of septation onset, cell size and anaphase B progression was investigated in detail. Both the time of septation onset and cell length were analyzed in short (cell cycle *wee1-50* and *cdc2-3W*), normal and long cells (wild-type diploid, cell cycle *cdc25-22* and *cdc10-119*) (Fig 2A–2E and Table 2), and a linear correlation was found between the two (Fig 2F). To determine if the start of septation correlates with a specific stage during anaphase B proportional to the cell size, the spindle length during anaphase B was measured. It was found that the percentage of the spindle length at septation onset as compared to the maximal spindle length at late anaphase B was similar in normal and long cells, but slightly reduced in short cells (Table 3). However, the percentage of elapsed time at septation onset during anaphase B compared to the total mitosis time was consistently similar in wild-type, long and short cells (Fig 2G and S1 Table). Overall, these results reveal that the timing of septation onset, with respect to anaphase B, scales with cell size, and support the existence of a cell size-dependent control that helps to coordinate septum and furrow formation with anaphase B progression.

**Table 3:** Spindle length at septation onset with respect to the cell size and the maximal spindle length.

Strain	Spindle length at septation onset^1^	Cell length^2^	Septation / cell size ratio^3^	Spindle length at late anaphase^4^	Septation / spindle length ratio^5^
**28°C**					
**Wild-type** (17 cells)	7.8 ± 0.7 μm	13.9 ± 0.8 μm	55.9 ± 3.8 (%)	10.8 ± 0.6 μm	71.3 ± 5.7 (%)
***cdc13-GFP*** (15 cells)	7.9 ± 0.9 μm	14.1 ± 1.0 μm	55.9 ± 4.7 (%)	11.3 ± 0.9 μm	69.9 ± 6.3 (%)
***cdc15-GFP*** (11 cells)	11.0 ± 0.6 μm	15.3 ± 1.1 μm	72.2 ± 5.9 (%)	11.4 ± 0.6 μm	96.4 ± 2.7 (%)
***sid2-250*** (11 cells)	10.6 ± 0.7 μm	15.2 ± 0.7 μm	70.0 ± 4.8 (%)	12.1 ± 0.7 μm	87.6 ± 7.2 (%)
**25°C**					
**Wild-type** (15 cells)	7.9 ± 0.9 μm	14.7 ± 0.8 μm	53.7 ± 5.9 (%)	11.5 ± 0.9 μm	69.0 ± 6.4 (%)
***cdc25-22*** (10 cells)	14.8 ± 1.6 μm	37.8 ± 3.9 μm	39.2 ± 3.6 (%)	21.1 ± 2.2 μm	70.4 ± 6.9 (%)
***cdc10-119*** (9 cells)	11.1 ± 0.7 μm	28.3 ± 3.5 μm	39.4 ± 2.6 (%)	17.6 ± 1.7 μm	64.3 ± 3.4 (%)
**Wild-type diploid** (16 cells)	12.2 ± 1.3 μm	21.5 ± 1.8 μm	57.2 ± 5.4 (%)	14.6 ± 0.8 μm	84.1 ± 7.8 (%)
***wee1-50*** (13 cells)	5.1 ± 0.7 μm	10.2 ± 0.8 μm	50.2 ± 6.1 (%)	8.7 ± 0.6 μm	58.3 ± 10.3 (%)
***cdc2-3W*** (17 cells)	5.2 ± 0.7 μm	9.3 ± 1.1 μm	55.6 ± 7.9 (%)	8.3 ± 1.1 μm	61.2 ± 9.4 (%)

Values are mean ± SD.

The distance between chromosome masses was measured in cells randomly selected from the experiments shown in Table 1 (28°C) and Table 2 (25°C).

^1^ Length between chromosome masses (= spindle length) at septation onset.

^2^ Cell length at septation onset.

^3^ Percentage of spindle length at septation onset with respect to the total cell length.

^4^ Maximal length between separated chromosome masses (= spindle length) at late anaphase.

^5^ Percentage of spindle length at septation onset with respect to the maximal spindle length at the end of anaphase.

**Fig 3:**
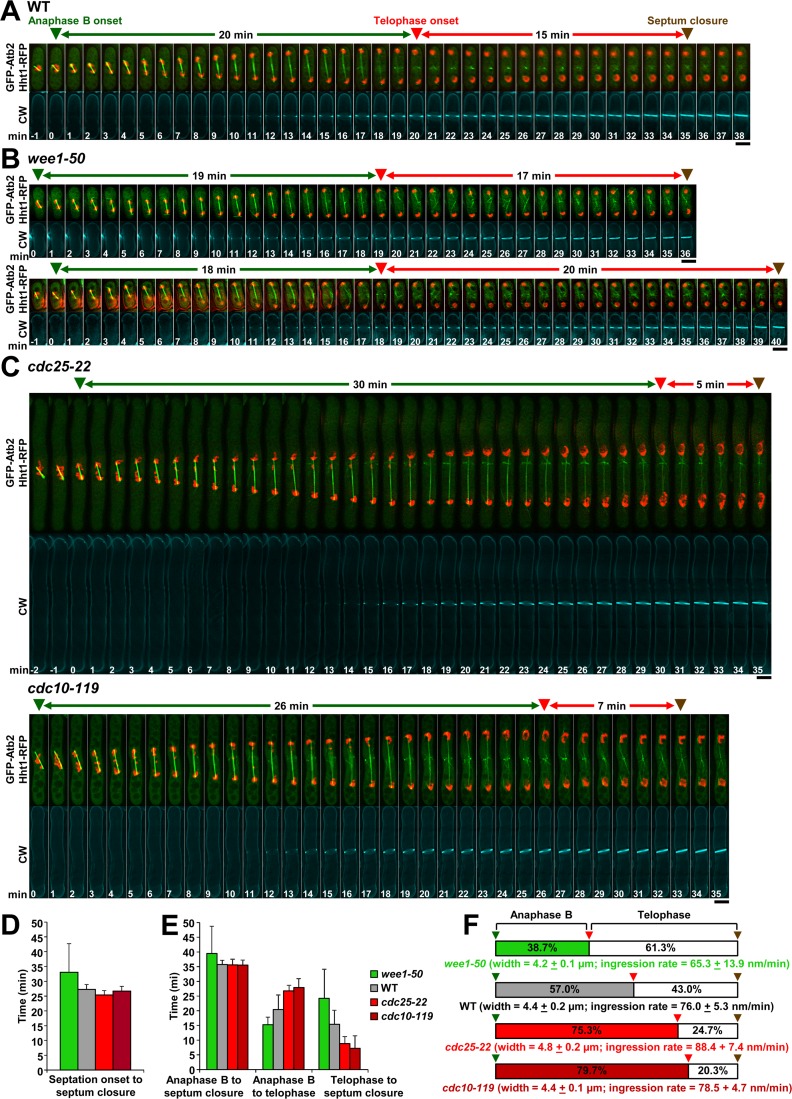
The elapsed time from telophase onset to septum closure correlates inversely with the cell size. Wild-type (n = 2, 10 cells) **(A)**, cell cycle-short *wee1-50* (n = 3, 10 cells) **(B)**, and cell cycle-long *cdc25-22* (n = 3, 10 cells) and *cdc10-119* (n = 2, 10 cells) **(C)** cells were grown as in Fig 2 and imaged as in Fig 1. **(D)** The elapsed time from septation onset to septum closure is similar in wild-type and long cells, and increased in short cells. **(E)** The elapsed time from telophase onset to septum closure is reduced in long cells and increased in short cells with respect to that of wild-type cells. Graphs show the elapsed time for the indicated intervals in the cells of A, B and C. Error bars indicate standard deviation (SD). **(F)** Scheme of the percentages of elapsed times shown in E. The cell width (μm ± SD) and rate of septum ingression (nm/min ± SD) for each strain are shown. Brown arrowhead, septum closure. Other symbols are as in Fig 1. Bars, 5 μm.

The timing of septum closure (brown arrowhead, Fig 3A–3C) with respect to the cell size was also studied. The elapsed times from either anaphase B onset or septation start to septum closure at telophase were similar in wild-type and long cells, but extended in small cells (Fig 3D and 3E). Interestingly, the elapsed time from telophase onset to septum closure was greatly reduced in long cells and increased in short cells, with respect to that of wild-type cells (Fig 3E and 3F). The close proximity between spindle disassembly and septum closure in long cells suggests that adjustments in the timing of septation onset during anaphase B might be required to avoid the breakage of a persistent mitotic spindle, thus ensuring that chromosomes are safely separated from the division site and the divided nuclei are properly centered in the newly formed cells.

### Septation onset depends on the inactivation of Cdk1 in fission yeast

**Fig 4:**
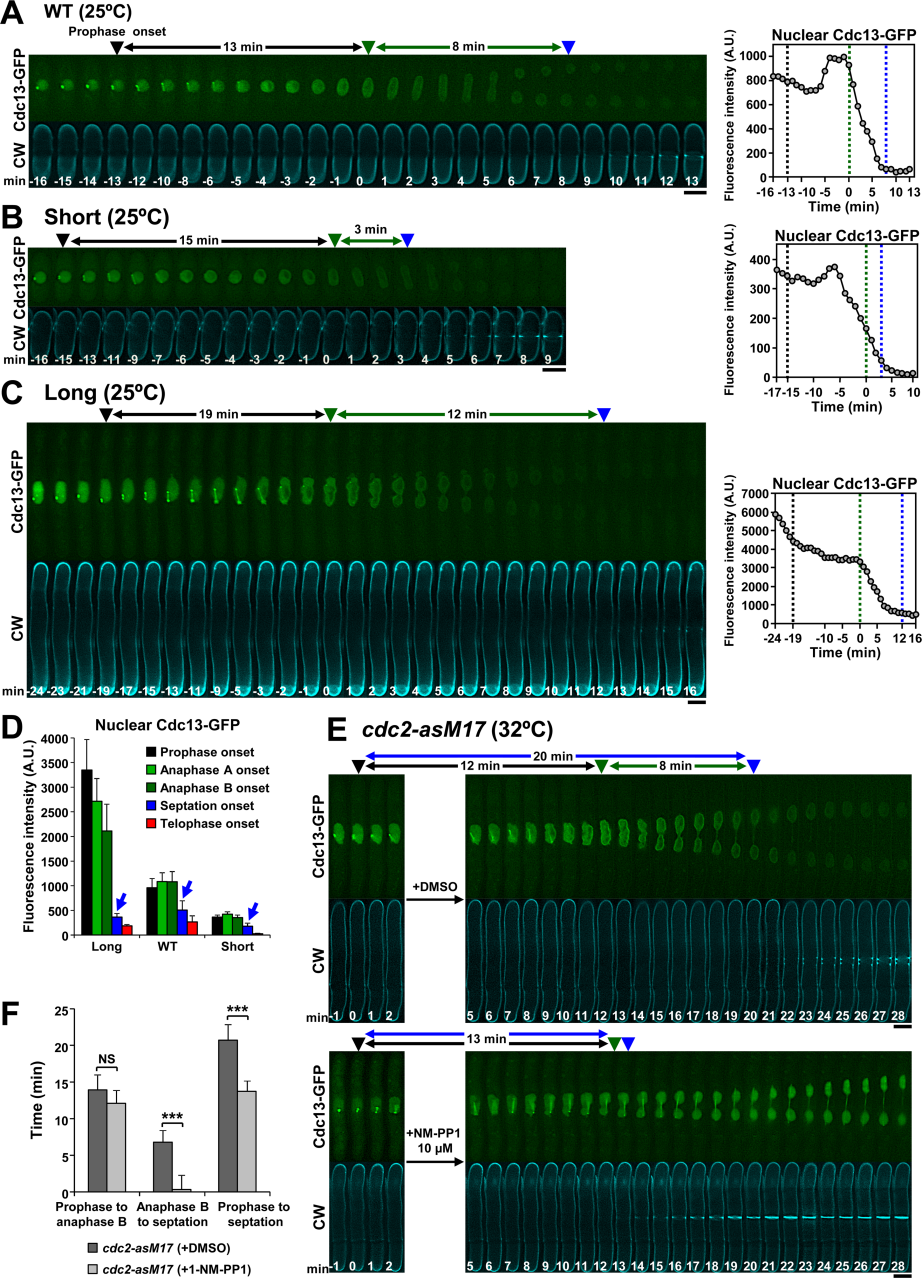
The onset of septation depends on the inactivation of Cdc2/Cdk1 kinase in fission yeast. **(A)** Septation start in wild-type cells coincides with the decline of Cdk1-associated cyclin B Cdc13. **(B)** Short cells display premature septation onset that coincides with the anaphase B onset and the decline of nuclear Cdc13. **(C)** The initiation of septum deposition is significantly delayed in long cells but coincides with the decline of nuclear Cdc13. Cells were grown in YES at 25°C (A and B) or released at 25°C after 3.5 h of cell cycle arrest at 36°C (C), and imaged as in Fig 1. Graphs to the right show the total fluorescence of Cdc13-GFP in the nuclei in the series to the left. Dashed lines in graphs correspond to the arrowheads in the series. A.U., arbitrary units. Nuclear Cdc13-GFP was quantified as described in the Material and Methods section. **(D)** The septation onset coincides with the decline of nuclear Cdc13 during early anaphase B. Graph showing the levels of nuclear Cdc13 fluorescence in the population of wild-type (n = 2, 14 cells), long (n = 2, 8 cells) and short cells (n = 2, 15 cells) in the indicated mitotic stages in the cells of A, B and C. Cells were grown and imaged as in Fig 1, and Cdc13 was quantified at the indicated stages as in A. Blue arrow indicates the levels of Cdc13 at septation onset. Error bars indicate standard deviation (SD). **(E, F)** Inactivation of Cdc2 kinase in early mitosis induces a very premature septation onset. (E) ATP-analogue sensitive *cdc2-asM17* mutant cells carrying Cdc13-GFP were G2-arrested by growth in the presence of 1 μM 1-NP-PP1 for 3.5 h at 32°C. Then, the cells were G2-released by transfer to a fresh medium and imaged to detect the entry into mitosis. Cdc2 was inactivated during early mitosis transferring the cells to a fresh medium containing either DMSO (control cells; n = 2, 14 cells) or 10 μM 1-NP-PP1 (cells with inactive Cdk1; n = 4, 37 cells). Cells were imaged at 32°C as in Fig 1. (F) Graph showing the elapsed time in the indicated mitotic intervals of cells in E. The asterisks indicate the significant statistical difference between paired strains analyzed by the Student’s test: * p <0.05; ** p <0.01; *** p <0.001; NS: not significant (p >0.05). Error bars indicate standard deviation (SD). Symbols are as in Fig 1. Bars, 5 μm.

The fact that septation starts before mitosis exit and very close in time to the described Cdc2/Cdk1 kinase inactivation at early anaphase B [32] prompted us to evaluate its direct dependence on Cdk1 activity. Thus, the role of cyclin Cdc13 location and decline in the septation onset was examined. Cdc13 localizes to the SPBs and spindle during prophase and metaphase, and moves to the nuclear periphery in anaphase A, where it is presumably degraded when the spindle elongates in anaphase B [33]. Septation onset coincided with the large drop of nuclear Cdc13-GFP fluorescence (Fig 4A and Fig 4D). In short cells the premature septation was coincident with the anaphase B onset and the complete decay of Cdc13-GFP (Fig 4B and Fig 4D). By contrast, long cells showed a slow decline of Cdc13-GFP and coincided with a large delay in the start of septation (Fig 4C and Fig 4D). Therefore, the timing of Cdk1 downregulation is dependent on the cell size like the timing of septation onset.

To test if septation onset specifically depends on Cdk1 inactivation we first analyzed the effect of a non-degradable Cdc13 in the timing of septation onset (S2 Fig). Expression of endogenous non-degradable Cdc13 [34] caused an anaphase B blockage as previously reported with an inducible version [7]. Two types of cells arrested in anaphase B were observed: Type 1 cells exhibited a considerably delayed septation and mild or non-apparent defects in chromosome segregation, and Type 2 cells exhibited blocked septation and persistent trailing chromosomes and/or failed chromosome segregation (S2A and S2B Fig).

Then we analyzed the timing of septation onset in cells with Cdk1 specifically inactivated at early mitosis. For this purpose, the analogue-sensitive *cdc2-asM17* mutant was used [35]. Specific Cdk1 inactivation by incubating *cdc2-asM17* cells with 10 μM 1-NP-PP1 immediately after their entry into mitosis, led to a premature septation onset (Fig 4E and 4F). These data show that Cdk1 inactivation is required for the immediate and accurate activation of septation during early anaphase B.

### The low levels of SIN activity at early anaphase B are enough to trigger the Sid2 relocation to the cell middle and the ensuing activation of septum synthesis

To further understand the mechanisms that trigger the start of septation during early anaphase B, we examined if the described requirements for septation during telophase are also required in early anaphase B. The chronological localization as a stable ring of the main cytokinetic components regarding septation start was examined. All the analyzed AR proteins sequentially coalesced during the 9 minutes prior to septation onset (S3A Fig and S3C Fig), while Bgs1 was detected as a stable ring 30 seconds before septation onset. Other GSs stably localized after septation start, Ags1 during late anaphase B and Bgs4 at telophase (S3B and S3C Fig), in agreement with the proposed role of Bgs1 and/or the PS determining the location of the rest of GSs [29]. As expected, the presence of a defective AR led to delayed septation onset (S3D and S3E Fig, Table 1 and Table 3). These results indicate that the AR present at early anaphase B is mature and ready to induce Bgs1 recruitment and immediate septation onset.

**Fig 5:**
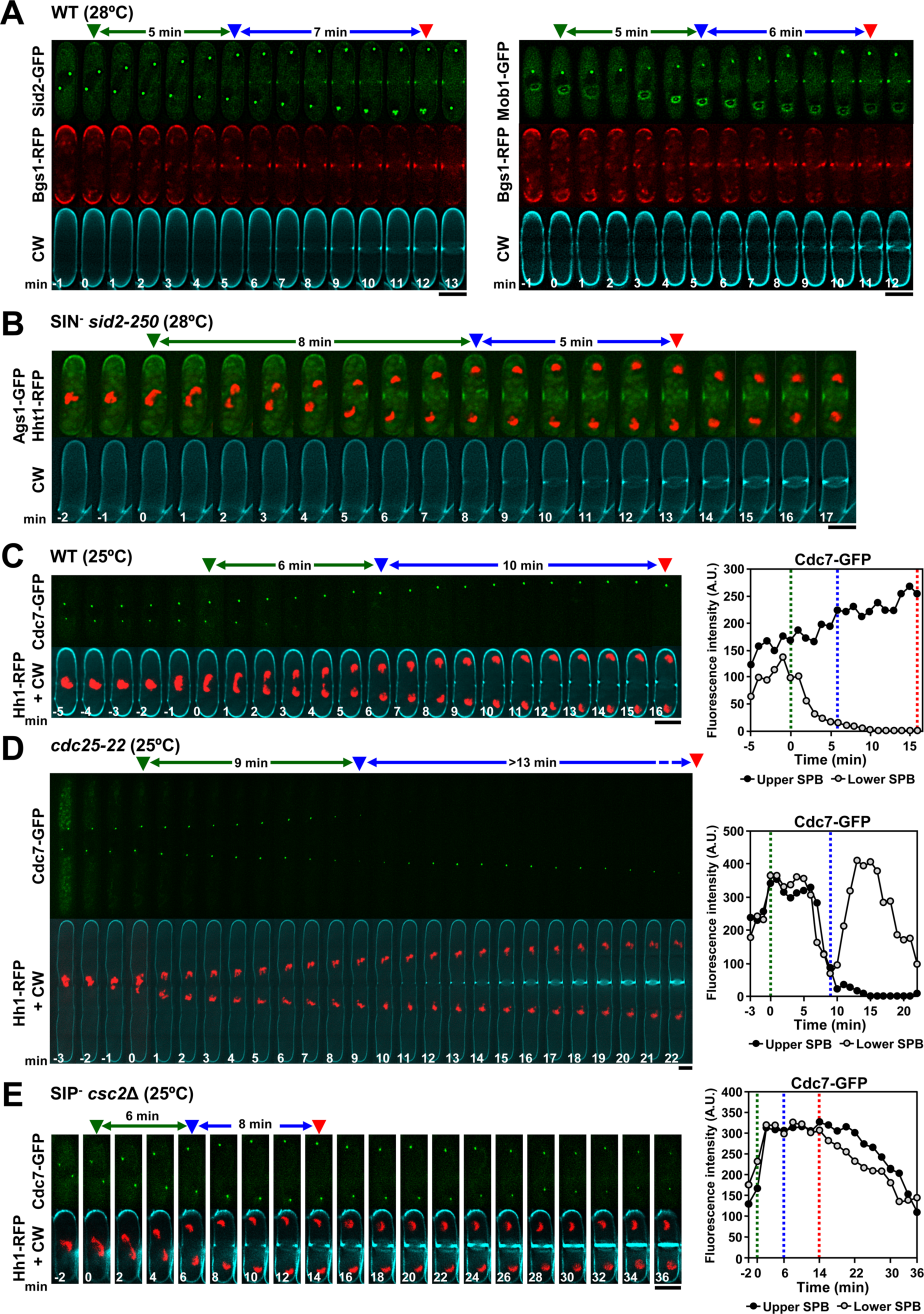
The activation of septum synthesis in early anaphase B depends on SIN function, but not on the asymmetric SIN in the SPB. **(A)** Sid2-Mob1 kinase complex localizes to the division site just after Bgs1 localization and coincident with the septation onset. Cells were grown and imaged as in Fig 1. **(B)** Septation start is delayed when the function of Sid2 is compromised. Cells were grown in YES at 25°C, shifted to 28°C for 4 h and imaged as in Fig 1. The data of cells of B are developed in Table 1 and Table 3. **(C, D)** Septation start coincides with the asymmetric disappearance of Cdc7 from one SPB. Cells were grown in YES at 25°C (wild-type, C) or released at 25°C after 3.5 h of cell cycle arrest at 36°C (*cdc25-22*, D), and imaged by time-lapse fluorescence microscopy (maximum-intensity projections of 7 z slices at 0.4 μm intervals for Cdc7-GFP and 1 medial z slice for CW-staining, 1 min elapsed time). **(E)** Timely activation of septum synthesis does not depend on SIN asymmetry. Defective SIN-Inhibitory Phosphatase (SIP) complex *csc2*Δ cells were examined as in C. The data of cells of C, D and E are developed in S2 Table. Graphs to the right show the total fluorescence of Cdc7-GFP in each SPB in the series to the left. Dashed lines in graphs correspond to the arrowheads in the series. A.U., arbitrary units. Symbols are as in Fig 1. Bars, 5 μm.

It is assumed that maximal SIN activity at late anaphase B triggers septation by promoting Sid2-Mob1 kinase complex relocation from the SPB to the AR [20, 36, 37]. We found that both Sid2-GFP and Mob1-GFP relocated much earlier than described to the cell middle, coinciding with the first detection of PS (Fig 5A, S3A Fig and S3C Fig) and suggesting that the SIN triggers septation before its full activation at late anaphase. Previous studies proposed that Bgs1 displacement to the cell middle requires the SIN signaling [38–40]. In contrast, it has been shown that Ags1 displacement to the cell middle does not require the SIN [27]. Thus, the GSs localization in the absence of SIN was reexamined. It was found that all GSs appeared as a broad medial band after the transient formation of the AR but never concentrated as a ring in SIN-defective *cdc11-119* cells (S4 Fig). These data show that proper localization of GSs as a ring depends on both SIN and AR, as previously described for Ags1 [27], and suggest that the SIN might trigger septation by maintaining the AR and regulating GSs functions.

Next, the role of SIN signaling PS formation at early anaphase B was analyzed. *sid2-250* mutant cells display reduced SIN even at the permissive temperature [18, 41]. We found that septation started significantly later in *sid2-250* cells (Fig 5B, Table 1 and Table 3), indicating the Sid2 dependence for septation start at early anaphase B, and that the still low level of SIN activity at early anaphase B is enough to trigger Sid2-Mob1 relocation and septation onset.

Cdc7 kinase levels at the SPBs are used as an indirect assessment of SIN activity [14]. Also, Cdc7 disappearance from one SPB is considered a hallmark for SIN signaling [42]. Therefore, the role of the SIN asymmetry was examined; detecting that septation start coincided with the total Cdc7 loss from one SPB (Fig 5C and S2 Table). It has been described that SIN asymmetry scales with cell size [43] and as expected, the timing of symmetric SIN increased in long *cdc25-22* cells, as did the timing of septation, matching the Cdc7 asymmetry (Fig 5D and S2 Table). To determine if the asymmetric SIN is required for the timely activation of septum synthesis, *csc2*Δ cells deprived of the SIN-inhibitory phosphatase complex (SIP), which is required for SIN asymmetry in anaphase B, were analyzed [44]. As expected, the SIN remained symmetric through anaphase B and telophase. However, the timing of PS detection was unaffected and occurred just after the SIN reached a maximum in both SPBs in early anaphase B (Fig 5E and S2 Table). Likewise, SIN asymmetry and septation onset did not correlate, either when the symmetry was extended at higher temperature, or when septation onset was delayed by a partially defective Bgs1 function (S5 Fig and S2 Table). All these observations indicate that the timely septation onset does not depend on the establishment of SIN asymmetry.

### The levels of SIN Etd1 and Rho1 GTPase regulate the timing of septation onset

The timing of septation onset depends on Cdk1 inactivation and the ensuing level of SIN activity during early anaphase B, and all three depend on the cell size. Etd1 is the only known SIN activator that localizes to cortex and cytoplasm at both the cell middle and tips, and its function is critical for SIN signaling [17–19, 43]. Thus cell middle-localized Etd1 should be the best SIN candidate to change its concentration and therefore the SIN activity with the cell size. In agreement, the activation of septum synthesis in cells with displaced SPBs and with Etd1 normally localized in the cell tips and middle did not depend on the proximity of the Cdc7-active SPB to the cell tip (S6A and S6B Fig). In addition, the Etd1 localization in medial cortex and cytoplasm did not depend on the nucleus position but on the AR and ensuing septum position (S6C Fig), as previously described [17].

**Fig 6:**
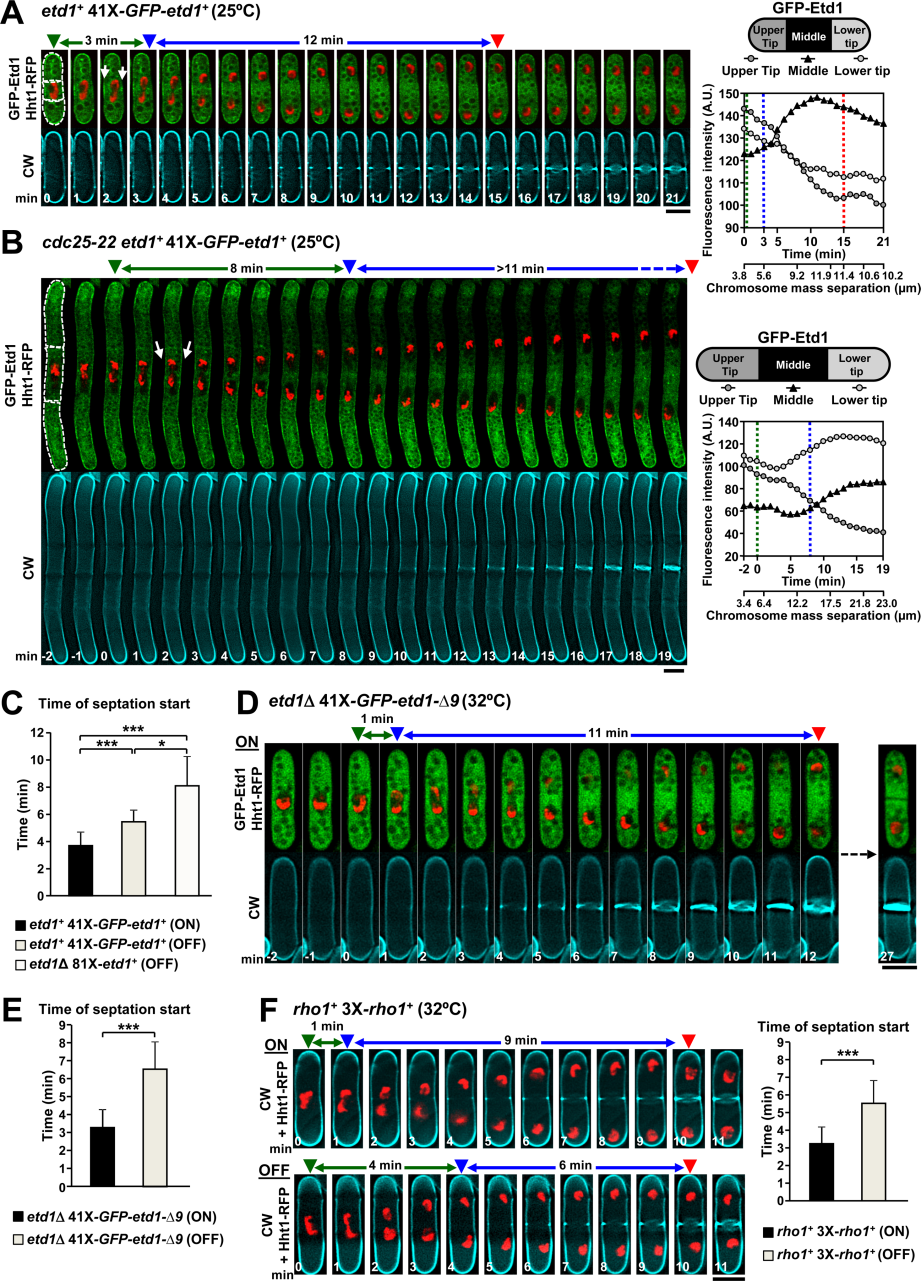
The levels of Etd1 and Rho1 regulate the timing of septation start. **(A)** The timing of septum deposition onset correlates with the start of increase of Etd1 in the cell middle. Cells were grown in MM without thiamine (*GFP-etd1*^*+*^ induced) at 25°C for 24 h and imaged as in Fig 1. **(B)** The increase of Etd1 in the cell middle and concomitant initiation of septation are delayed in long cells. Cells were analyzed as in A after 3.5 h of cell cycle arrest at 36°C. Graphs to the right show the total fluorescence of GFP-Etd1 at the cell poles and middle in the series to the left. A.U., arbitrary units. Arrow, cortical localization of Etd1 in the cell middle. Dashed outlines indicate the ROIs used to measure the total fluorescence of GFP-Etd1 in the corresponding regions of the cell. **(C)** The timing of septation onset is dependent on the level of *etd1*^*+*^. Cells expressing endogenous *etd1*^*+*^ and 41X*-GFP-etd1*^*+*^ grown at 32°C for 24 h either in the absence (ON, high *etd1*^*+*^ level; n = 4, 34 cells) or in the presence of thiamine (OFF, wild-type *etd1*^*+*^ level; n = 2, 11 cells), and cells expressing *etd1*Δ 81X*-etd1*^*+*^ grown for 15 h with thiamine (OFF, very low *etd1*^*+*^ level; n = 3, 23 cells), just before the emergence of SIN phenotype were analyzed as in A. **(D, E)** The start of septum synthesis does not dependent on cortical Etd1. Cells expressing a functional Etd1 version that is absent from the cortex from *etd1*Δ 41X-*GFP-etd1*-∆*9* strain grown at 32°C either for 6 to 9 h without thiamine (ON; n = 2, 37 cells) or with thiamine (OFF; n = 2, 17 cells) were analyzed as in A. **(F)** The start of septum deposition is dependent on the level of *rho1*^*+*^. Cells expressing endogenous *rho1*^*+*^ and 3X*-rho1*^*+*^ grown at 32°C for 16 h either without (ON, high *rho1*^*+*^ level; n = 2, 14 cells) or with thiamine (OFF, wild-type *rho1*^*+*^ level; n = 2, 16 cells) were analyzed as in A. The asterisks in C, E and F indicate the significant statistical difference between paired strains analyzed by the Student’s test: * p <0.05; ** p <0.01; *** p <0.001; NS: not significant (p >0.05). Symbols are as in Fig 1. Bars, 5 μm.

In order to know the mechanisms that regulate the SIN to trigger septation during early anaphase B depending on the cell size, the location and levels of GFP-Etd1 at the onset of septation were investigated. At anaphase B start, Etd1 localized to both cytoplasm and cortex of the cell middle (arrow, Fig 6A) and cell tips. Interestingly, PS synthesis initiated when the Etd1 levels started to increase in the cell middle cytoplasm, while gradually disappeared from the cell cortex (Fig 6A). Etd1 was not observed either like a defined ring or in the septum membrane throughout anaphase B, instead it concentrated along the membrane of advanced septa at telophase (Fig 6A and S7 Fig), supporting the idea that Etd1 signals SIN activation from the cytoplasm surrounding the SPBs. Like septation onset, the SIN activity during early anaphase B depends on the cell size [43]. In agreement, the increase of Etd1 levels in the cell middle was delayed in long *cdc25-22* cells, and the increase start coincided with the septation onset (Fig 6B).

To see if Etd1 levels in the cell middle regulate the start of septation, the timing of septation relative to different levels of Etd1, including increased (induced 41X-*etd1*^*+*^) and reduced Etd1 levels (repressed 81X*-etd1*^*+*^), was examined. Septation was initiated considerably earlier in Etd1-overproducing cells and was greatly delayed in Etd1-depleted cells (Fig 6C). These results show that Etd1 levels regulate septation onset, which is likely activated by the increase of cell middle Etd1 in early anaphase B.

Finally, to test whether the cortex-localized Etd1 is required for septation onset, the timing of septation onset was analyzed in cells expressing a functional Etd1 version that is absent from the cortex and exclusively localizes in the cytoplasm [19]. Similar to wild-type Etd1, the overproduction of GFP-Etd1-Δ9 greatly advanced the start of PS synthesis (Fig 6D and 6E). These results indicate that the start of septation exclusively depends on cytoplasmic Etd1.

SIN presumably triggers septum synthesis by regulating the activity of the GTPase Rho1 [21], which is the regulatory subunit of the GS [45]. In agreement, we observed that Rho1 overproduction (induced 3X-*rho1*^*+*^) advanced the septation start (Fig 6F). Altogether, these results indicate that the precise initiation of septation relative to the cell size depends on the levels of cytoplasmic Etd1 and Rho1, and suggest that both are part of a molecular mechanism coordinating septum and furrow formation with cell size during anaphase B.

### The mitotic spindle is required to restrain SIN signaling and septation onset during early anaphase B in fission yeast

**Fig 7:**
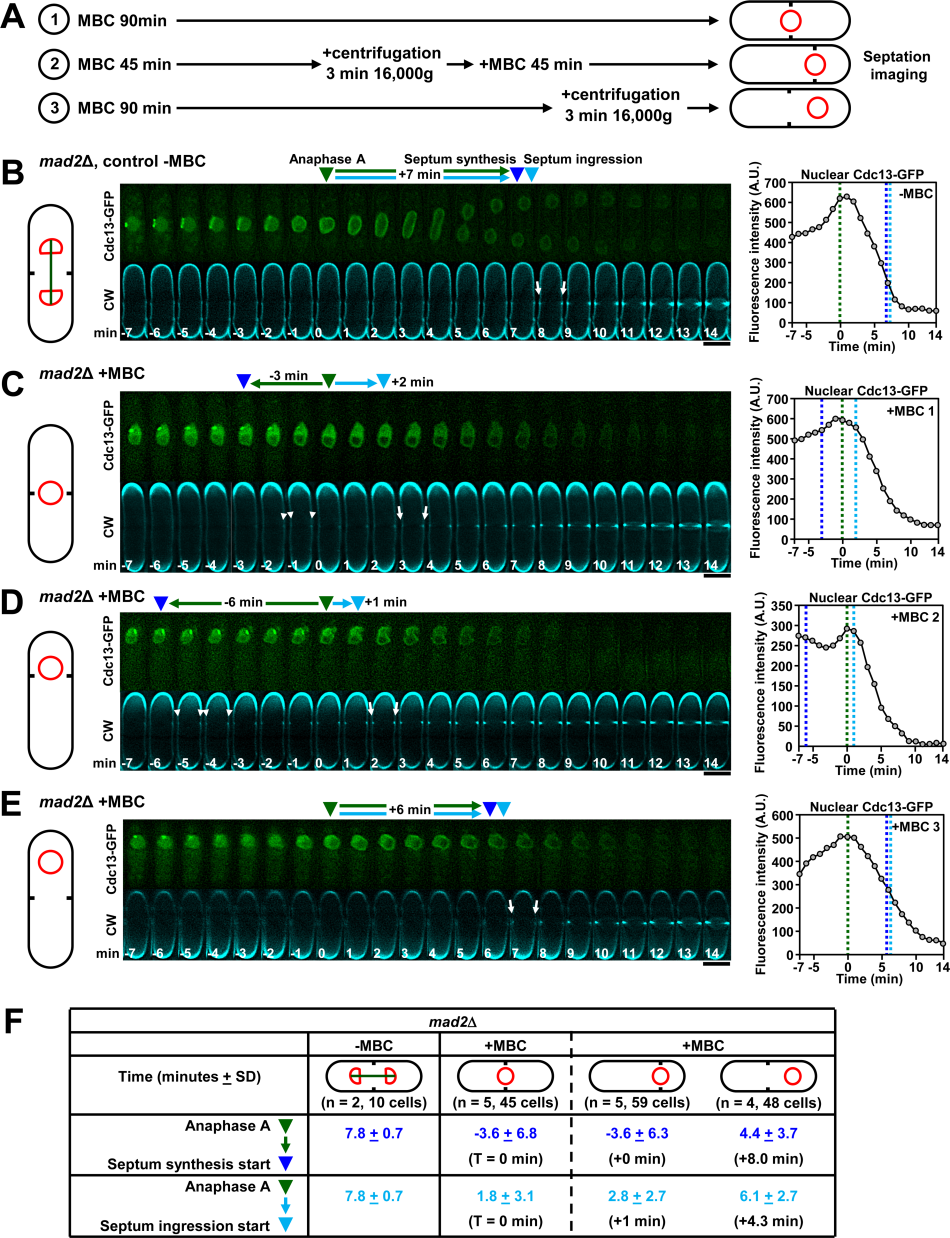
The spindle and the proximity of the nucleus to the division site are required for proper septum synthesis activation in fission yeast. **(A)** Scheme of the steps required to prevent nuclear separation and to maintain or separate the undivided nucleus from the cell middle and/or from the division site. (A-1 and **C**) Nucleus and division site are maintained in the cell middle; cells were treated for 90 min and imaged with methyl 2-benzimidazolecarbamate (or carbendazim, MBC, 50 μg ml^-1^) to avoid spindle assembly and nuclear separation. (A-2 and **D**) Nucleus and division site are relocated to a cell end; cells were treated for 45 min, centrifuged to displace the nucleus, treated 45 more min and visualized with MBC. (A-3 and **E**) The nucleus is relocated and separated from the division plane; cells were treated for 90 min, centrifuged and examined with MBC. **(B)** *mad2*Δ cells were grown and imaged without MBC as in Fig 1. The *mad2*Δ cells were used to avoid a delay caused by the activation of the spindle assembly checkpoint. **(C-E)** The premature and uncoupled septation start caused by the absence of the spindle depends on the position of the nucleus. *mad2*Δ cells were processed as in A to prevent nuclear separation and to maintain or separate the undivided nucleus from the cell middle and/or the division site. (C) The nucleus and division site are maintained in the cell middle. (F) The nucleus and division site are relocated to a cell end. (E) The nucleus is relocated and separated from the division plane. MBC-treated cells were imaged as in B. Anaphase A onset was considered as time zero. Graphs to the right are as in Fig 4. Dashed lines and arrowheads: green, anaphase A onset; dark blue, septum synthesis start; light blue, septum ingression onset. White arrowhead: first CW-stained septum synthesis detection. White arrow: first CW-staining increase showing septum ingression. A.U., arbitrary units. **(F)** Uncoupled septum synthesis and ingression timing with MBC is restored to wild-type levels when the undivided nucleus is separated from the division site. Table showing the time between anaphase A (green) and septum synthesis start (dark blue) or septum ingression onset (light blue) in the indicated cells. Parenthesis: n, number of experiments and cells; T, delay in septum synthesis and ingression start with respect to control cells with MBC as in C. Bars, 5 μm.

Spindle elongation is also required for SIN signaling during anaphase B [18]. Therefore we tested whether the spindle is required for timely septation onset by examining the cells with the MT-depolymerizing drug carbendazim (MBC, Fig 7A). To avoid a delay caused by activation of the spindle assembly checkpoint, a strain lacking Mad2 was used [46]. Septation onset was not affected in MBC-untreated *mad2*Δ cells and coincided with the large decline of Cdc13 cyclin (Fig 7B and Fig 7F). Surprisingly, the spindle absence caused by MBC treatment induced a highly premature septation, while the cells still maintaining high levels of Cdc13, and with the PS initiation uncoupled (CW fluorescence remains weak and small) from the ensuing septum ingression (CW fluorescence starts to increase) (Fig 7C and Fig 7F). These data suggest that septation is negatively regulated by the early-mitosis spindle preventing a premature Cdk1-independent septation activation before chromosome mass separation.

Coinciding with the assembly of the mitotic spindle at prophase onset, the SPBs are specifically embedded in the nuclear envelope until the anaphase B onset, where both SPBs are extruded to the cytoplasm [47]. Given that the forces exerted by the elongating spindle might be required for both the SPBs insertion in the membrane and the nuclear envelope reinforcement [48], to test if the advanced septation onset in the absence of spindle could be caused by an altered SPB localization, cells carrying nucleoporin Cut11-GFP were analyzed (S8A Fig). Nucleoporin Cut11 specifically localizes to the SPBs but only when embedded at the nuclear envelope from prophase to anaphase B onset [49]. In control cells the start of both PS synthesis and septum ingression was coupled and it was coincident with the SPBs exit and Cut11 loss (S8A Fig). However, during premature onset of PS synthesis without spindle, Cut11 always localized to the SPBs. Interestingly, septum ingression was uncoupled and delayed from PS synthesis onset until the loss of SPB-located Cut11 (S8A Fig). These results suggest that the SPBs play a role in the septation onset and that nuclear envelope-embedded SPBs might help to prevent the start of septum ingression in early mitosis.

The spindle regulation of cytokinesis might also depend on the nucleus/SPBs position, regarding the division site and therefore the Etd1 medial localization. Thus, septation in cells devoid of MT and with the nucleus displaced to the pole, both before and after division site selection, was examined (Fig 7A2 and Fig 7A3) [50]. When the nucleus, division site and Etd1 were displaced in the absence of MT, septation advanced to a similar timing as that of nucleus-centered MBC-treated cells (Fig 7D, Fig 7F and S8B Fig). In contrast, when the nucleus was displaced, but the division site and Etd1 remained in the middle, septation was noticeably delayed (Fig 7E, Fig 7F and S8B Fig). These results indicate that the nucleus position near the division site and Etd1 is required for the premature septation onset caused by the absence of spindle.

**Fig 8:**
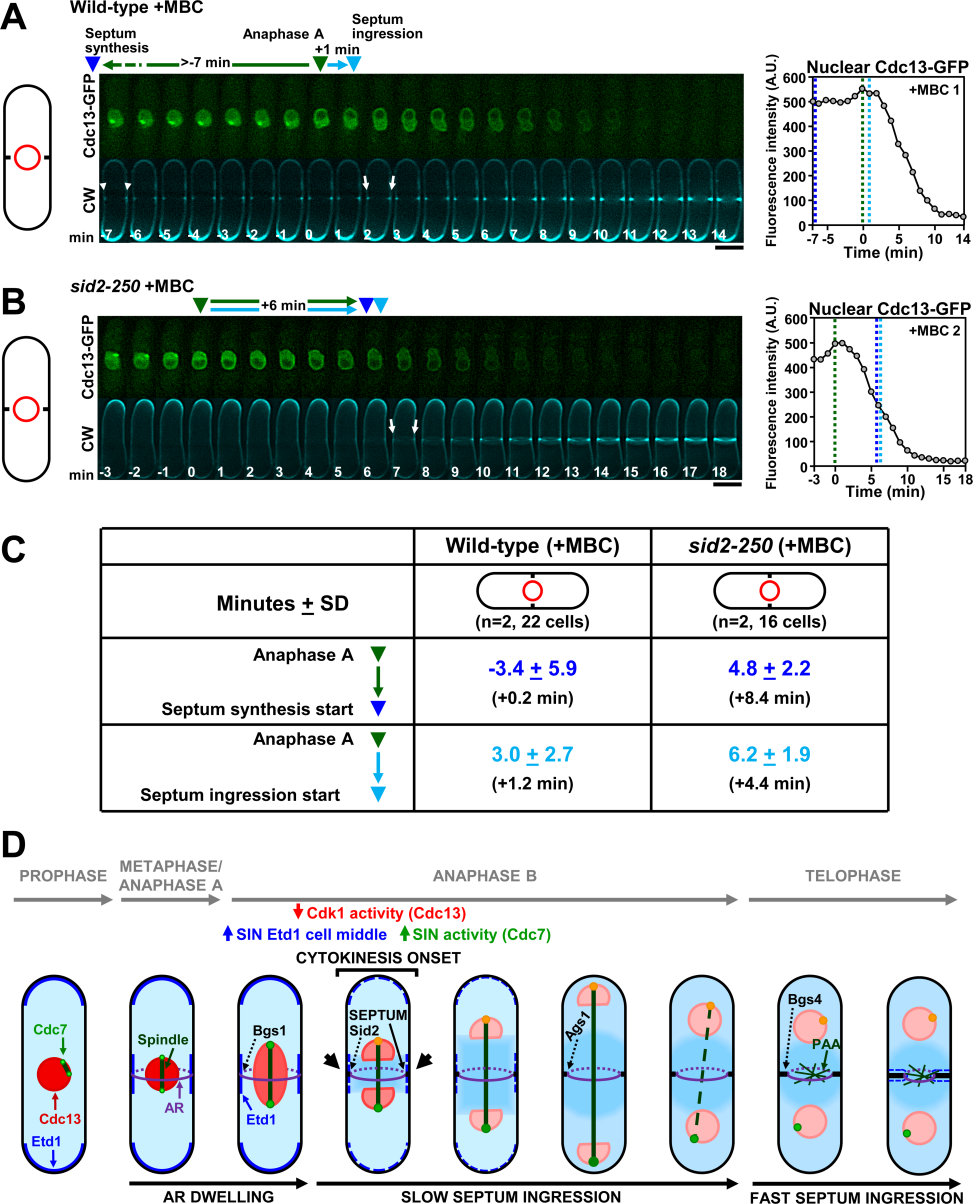
The premature and uncoupled septation start caused by the absence of spindle MT is dependent on the SIN pathway but not on the spindle assembly checkpoint. **(A)** The absence of spindle MT induces a dramatically premature and uncoupled septum synthesis start and ingression onset even in the presence of the spindle assembly checkpoint. Wild-type cells containing *mad2*^+^ gene were processed with MBC as described in Fig 7C to avoid spindle assembly and nuclear separation. **(B)** The premature septum synthesis initiation of A (also compare with values in Fig 7F) depends on the SIN. *sid2-250* cells grown at 28°C for 3 h were treated with MBC as in Fig 7C. **(C)** Uncoupled timings of septum synthesis and septum ingression in the presence of MBC are restored to wild-type levels when the SIN activity is affected. Table showing the time between anaphase A (green) and septum synthesis start (dark blue) or septum ingression onset (light blue) in the indicated cells. Symbols and values are as in Fig 7. A.U., arbitrary units. Bars, 5 μm. **(D)** Model for activation of septum and cleavage furrow start during early anaphase B. At early anaphase B, inactivation of Cdk1 (red, cyclin Cdc13) allows increase of SIN signaling (green, SIN Cdc7 of SPB) by enrichment of the SIN activator Etd1 (blue) in the cell middle. Initial SIN activation triggers Sid2 relocation to the division site and activation PS synthesis start by Bgs1 GS. Septum ingression is slow during anaphase B, and greatly increases at telophase onset coinciding with the spindle disassembly, the full SIN activation and the stable localization of Ags1 and Bgs4 GSs to the septum edge membrane.

Finally, to test if the advanced septation of MBC-treated cells depended on the spindle assembly checkpoint and/or the SIN signaling, the timing of septation was analyzed in wild-type and *sid2-250* cells treated with MBC. Wild-type cells exhibited the same premature and uncoupled activation of septum synthesis as that of MBC-treated *mad2*Δ cells without spindle (Fig 8A and Fig 8C). Contrary, the uncoupled timings of septum synthesis and septum ingression in the presence of MBC were restored to wild-type levels in *sid2-250* cells with reduced SIN (Fig 8B and 8C). Globally, these results show that the spindle elongation is required for the proper timely activation of septum deposition by negatively regulating the SIN signaling during early mitosis.

## Discussion

### The onset of septum and cleavage furrow ingression is activated at early anaphase B in fission yeast

The current cytokinesis model establishes that full SIN activation at late anaphase B triggers septation and AR closure onset after spindle breakage in telophase [9, 25]. However, analysis of cells stained with CW, which specifically binds to the septum L-BG [26], uncovered that PS synthesis begins at early anaphase B, long before the chromosome masses arrive at the cell tips and the spindle breaks down. Electron microscopy of early anaphase B cells revealed the presence of nascent PS growing inwards, which implies membrane invagination and the closure of the AR located to the new septum edge. In agreement, here we show that the septum is able to slowly ingress during anaphase B. Interestingly, the rate of septum ingression accelerates by 3-fold at telophase onset, which is coincident with the spindle disassembly, the complete activation of the SIN, and the stabilization of Ags1 and Bgs4 GSs in the septum edge (Fig 8D). Given that these GSs are responsible for the synthesis of the major septum polysaccharides α(1,3)glucan and branched-β(1,3)glucan [27, 28], their presence at the division site might be the cause of the increased rate of septum synthesis.

### Temporal control of septum synthesis activation in fission yeast

The SIN is already active in early mitosis [51–53], bringing into question why it would be kept from activating septation until telophase. The SIN displays two separable states during mitosis [43]. The ‘early’ state depends on Plo1 activity, and is characterized by the weak and unstable Cdc7 association with both SPBs. At early anaphase B the SIN changes to the ‘late’ state that is Plo1 independent and characterized by the increase of activity and asymmetric Cdc7 localization. This SIN ‘late’ state requires Cdk1 inactivation and scales with cell size [15, 43, 54]. Additionally, in this “late” stage there is a peak in Sid2 activity that coincides with the establishment of asymmetric SIN signaling [42], and that is also required for Etd1 translocation from the cell cortex to the cytoplasm during anaphase B [17, 18]. The transition between SIN states coincides temporally with the new timing of septation onset described in this work. Hence, the current model of cytokinesis activation was reevaluated (Fig 8D). After Cdk1 inactivation at early anaphase B, activation of the ‘late’ state reflects the SIN signaling for septum deposition by inducing Sid2 relocation to the division site. Strikingly, cells lacking the function of SIP complex, which is required for SIN asymmetry establishment [44], showed that this asymmetry is dispensable for the timely start of septation, but it could serve, together with the asymmetric Etd1 localization as an early preparatory event for the correct SIN inactivation at the end of cytokinesis [18, 44].

### Spatial control of septum synthesis initiation in fission yeast

Our study indicates that timely activation of septum synthesis requires an increase of Etd1 in the cell middle. In agreement, we found that the nucleus position with respect to the division site and cell middle Etd1 regulates the activation of septum synthesis. An intriguing hypothesis is that the early anaphase B spindle provides an equatorial zone free of chromosomes and a minimal spacing between dividing nuclei. Thus, in the absence of spindle, the SPBs would be overexposed to Etd1 in the cell middle, which might induce an excessive activation of the SIN and consequently, a premature septation. Similarly, in bacteria the nucleoid occlusion inhibitory system is required to prevent FtsZ-ring assembly and inappropriate cytokinesis without chromosome segregation [55]. In metazoans the specific length of the metaphase or early anaphase spindle grants the minimal space required by astral MT to induce the cleavage furrow onset [56]. However, the role of spindle and astral MT in the regulation of furrow ingression is controversial, with opposing results among the different organisms [57–59].

The absence of spindle could also alter SIN signaling through the function of the γ-tubulin complex, which might be important for the function and organization of the SPBs. This complex localizes to the SPB outer and inner faces, and is responsible for MT nucleation and anchorage [47, 60]. Thus, the spindle absence in the presence of MBC could also affect the function and/or location of the γ-tubulin complex, leading to changes in the spatial and temporal location of SIN kinases at the SPBs during early mitosis and thus, causing premature SIN signaling and septation start in a Cdk1-independent manner. In agreement, cells expressing a mutant version of the γ-tubulin complex also display premature septation with Cdc13 still present in the nucleus, and it was proposed that the SPB-associated γ-tubulin complex is required to inhibit premature SIN activation until Cdc13 is degraded [61].

### Size-dependent scalability of septum and furrow ingression initiation in fission yeast

Cells have different mechanisms to ensure adequate chromosome segregation in the daughter cells. Structural mechanisms of mitosis and cell division such as spindle elongation rate, spindle length, nuclear size [31, 56, 62] and AR closure rate [63, 64], as well as cell cycle signaling and transition to the SIN ‘late’ state [43, 65], scale in a size-dependent manner. Here we show a size-dependent scalability of septation start, coordinating cytokinesis with anaphase B and cell size.

Thus, timely septation depends on Cdk1 inactivation and Etd1 levels in the cell middle. Presently it is unknown how the release of Etd1 from the cell middle cortex affects its function, although there are evidences that strongly suggest that Etd1 regulates SIN signaling by interacting with cytoplasmic Spg1. Consistently, here we show that moderate overproduction of an Etd1 version that exclusively localizes to the cytoplasm leads to advanced septation start. Thus, the fact that Etd1 presumably activates the SIN from the cytoplasm near the SPBs is consistent with the Etd1 function being modulated in a size-dependent manner.

It is generally believed that septum and cleavage furrow, as determined by fluorescence microscopy, start to ingress after telophase onset [1–3, 25]. However, our study has established the precise size-dependent activation of septum synthesis and furrow formation during early anaphase B in fission yeast, providing new insights into the mechanisms that coordinate cytokinesis with mitosis to safeguard the chromosome segregation and proper nucleus placement in the newly developing daughter cells.

## Materials and methods

### Strains, growth conditions, and genetic methods

The *Schizosaccharomyces pombe* strains used in this study are listed in S3 Table.

Strains *GFP-bgs1*^*+*^ (1723), *GFP-bgs4*^*+*^ (2365), and *ags1*^*+*^*-GFP* (3166) contain the *bgs1*Δ::*ura4*^*+*^, *bgs4*Δ::*ura4*^*+*^ and *ags1*Δ 3’UTR_*ags1*+_::*ags1*_*3704-7233*_:*ura4*^*+*^ deletions and an integrated copy of SmaI-cut pJK-*GFP-12A-bgs1*^*+*^, StuI-cut pJK-*GFP-12A-bgs4*^*+*^ and AgeI-cut pJK-*ags1*_*1-6267*_*-12A-GFP-12A* (*leu1*^*+*^ selection), which direct their integrations at the SmaI site of the *bgs1*^*+*^ promoter sequence (nt -748) adjacent to *bgs1*Δ::*ura4*^*+*^, the StuI site of the *bgs4*^*+*^ promoter sequence (nt -1320) adjacent to *bgs4*Δ::*ura4*^*+*^ and the AgeI site of the *ags1*^*+*^ coding sequence (nt 6025) in *ags1*Δ 3’UTR_*ags1*+_::*ags1*_*3704-7233*_:*ura4*^*+*^, respectively. *2xGFP-bgs4*^+^ strain 2285 was made as *GFP-bgs4*^*+*^ strain, and contains an integrated copy of StuI-cut pJK-*2xGFP-12A-bgs4*^+^ (*leu1*^*+*^ selection), which directs its integration at the StuI site adjacent to *bgs4*Δ::*ura4*^*+*^, at position -1320 of the *bgs4*^*+*^ promoter sequence. Likewise, *2xRFP-bgs1*^+^ strain 1781 contains an integrated copy of SmaI-cut pJK-*tdTom-12A-bgs1*^+^ (tdTomato RFP variant, [66]) at position -748 of the *bgs1*^*+*^ promoter sequence.

Strains 5727 and 5728 were constructed by transforming the strain *cdc13*Δ::*ura4*^*+*^ P*nmt1*^*+*^*-*45-*cdc13*^+^:*sup3-5* (*sup3-5* selection marker recues *ade6-704* auxotrophy) [67] with an additional copy of either pJK-*cdc13*^*+*^ or pJK-*cdc13*^*des2*^ (*leu1*^*+*^ selection, native P*cdc13*^+^ promoter in both plasmids) respectively, and single-copy integrants were isolated. Next, strains 5735 and 5736 were made by a genetic cross between the strains described above and *hht1*^*+*^*-RFP*:*KanMX6* (5657), followed by random spore analysis selecting against the corresponding parental auxotrophies. Strains 5727 and 5735 exhibited wild-type phenotype either in the absence (-T, 45-*cdc13*^+^ and additional *cdc13*^+^ induced) or in the presence (+T, 45-*cdc13*^+^ repressed and additional *cdc13*^+^ induced) of thiamine. Strains 5728 and 5736 exhibited wild-type phenotype in the absence (-T, 45-*cdc13*^+^ and *cdc13*^*des2*^ induced), whereas in the presence of thiamine (+T, 45-*cdc13*^+^ repressed and *cdc13*^*des2*^ induced) the cells arrested in anaphase B, as described before for cells expressing *cdc13*^*des2*^ from an inducible promoter [7]. Western blot analysis of the strains grown in the presence of thiamine (+T, 45-*cdc13*^+^ repressed and additional *cdc13*^+^ or *cdc13*^*des2*^ induced) showed similar Cdc13 or Cdc13^des2^ protein levels.

Standard *S*. *pombe* rich (YES) and minimal (EMM) media, and genetic manipulations were used [68]. EMM with the appropriate supplements was used for strains expressing genes under the control of the thiamine-repressible P*nmt1*^+^ promoter. For repression experiments, early log-phase cells grown in EMM were diluted in the same medium and 20 μg ml^-1^ thiamine was added. SPA medium was used for genetic crosses and mutant strains were selected by tetrad dissection, random spore dissection or random spore analysis methods. Cell growth was monitored by measuring the A_600_ of early log-phase cell cultures.

Carbendazim or methyl 2-benzimidazolecarbamate (MBC, Sigma-Aldrich) was used at 50 μg ml^-1^ final concentration (from a stock of 5 mg ml^-1^ in DMSO, stored at -20°C). 4-Amino-1-tert-butyl-3-(1’-naphthylmethyl)pyrazolo[3,4-d]pyrimidine (1-NP-PP1, from a stock of 1 mM in DMSO, stored at -20°C, Toronto Research Chemicals Inc) was added to the media at the concentration of 1 μM to arrest the cells in G2 phase or 10 μM to quickly inactivate Cdc2 kinase during early mitosis.

### Plasmids and recombinant DNA methods

Plasmids pJK-*GFP-12A-bgs1*^*+*^, pJK-*GFP-12A-bgs4*^*+*^ and pJK-*ags1*_*1-6267*_*-12A-GFP-12A* are the integrative plasmid pJK148 (*leu1*^+^ selection) with a 9.6 kb ApaI-SpeI *GFP-12A-bgs1*^*+*^, 9.6 kb PstI-NheI *GFP-12A-bgs4*^*+*^, and 9.9 kb EcoRI-NheI *ags1*_*1-6267*_*-12A-GFP-12A* fragment, respectively. pJK-*2xGFP-bgs4*^*+*^ contains a 10.3 kb *2xGFP-12A-bgs4*^*+*^ fragment with a 1.5 kb tandem of two *GFP-12A* sequences cloned in-frame and separated by a 12-alanine linker to make a more flexible 2xGFP epitope. pJK-*tdTom-12A-bgs1*^*+*^ contains a 10.2 kb *2xRFP-12A-bgs1*^*+*^ fragment with the 1.4 kb tandem dimer tdTomato variant of the monomeric mRFP1 protein [66] (provided by R. Y. Tsien, University of California, La Jolla, CA) separated by a 12-alanine linker.

pJK-*cdc13*^*+*^ and pJK-*cdc13*^*des2*^ [34] are the integrative plasmid pJK148 (*leu1*^+^ selection) with a 4.2 kb KpnI-BamHI *cdc13*^*+*^ and *cdc13*^*des2*^ fragment respectively, containing the 1.4 kb coding sequence, a 1.9 kb promoter sequence and a 0.9 kb terminator sequence. The second destruction box mutant *cdc13*^*des2*^ was built by changing the *cdc13*^*+*^ coding sequence of amino acids RHALDDVSN to AHAADDVSN as described [7].

All DNA manipulations were carried out by established methods [69]. Enzymes were used according to the recommendations of the suppliers. Plasmid DNA was introduced into *S*. *pombe* cells by an improved LiAc method [70]. *Escherichia coli* DH10B was used as host to propagate plasmids by growth in Luria-Bertani medium containing 50 μg ml^-1^ ampicillin.

### Microscopy techniques and data analysis

Calcofluor white (CW) labeling for fluorescence images of cell cultures was performed adding directly a solution of CW (50 μg ml^-1^ final concentration, from a stock of 10 mg ml^-1^ in water) to early logarithmic phase cells. The CW is a fluorochrome that displays a high affinity for chitin, cellulose and L-BG. The fission yeast cells, which do not have either chitin or cellulose in their cell wall but contain L-BG in their PS, are extremely resistant to CW, growing in the presence of concentrations of up to 1.5 mg ml^-1^ of the dye [26, 71]. Images were obtained with a Leica DM RXA fluorescence microscope, a PL APO 63×/1.32 OIL PH3 objective, a Leica DFC350FX digital camera and Leica CW4000 cytoFISH software. Images were processed with the Adobe Photoshop software.

To follow the synthesis of the PS by time-lapse fluorescence microscopy, the cells were stained with a highly reduced concentration of CW (5 μg ml^-1^), which does not disturb the physiology of fission yeast cell growth and cytokinesis [27, 28]. To avoid unexpected cell effects due to the near-UV light exposure during image acquisition of CW-stained cells, the percentage of light transmission and the exposure time were greatly reduced. Similarly, since time-lapse fluorescence microscopy of multiple z-slices might occasionally alter the timing of septation onset of cells containing certain tagged proteins (data not depicted); time-lapses of one single medial z-slice were performed. When necessary, multiple z-slices were made under safe condition in which the septum synthesis start and septum ingression were not altered compared to the same processes observed in single z-slice time-lapses. Similarly, to reduce cellular stress, the time-lapse image acquisition was performed in liquid medium-containing chambers with cells growing freely only attached to the surface with a lectin, instead of using agarose pads with embed and immobilize cells. Early logarithmic phase cells (0.3–0.6 ml) were collected by centrifugation (1,000 g, 1 min) and resuspended in 0.3 ml of the same growth liquid medium containing CW (5 μg ml^-1^), and placed in a well from a μ-Slide 8 well or a μ-Slide 8 well glass bottom (80821-Uncoated and 80827; Ibidi) previously coated with 5 μl of 1 mg ml^-1^ soybean lectin (L1395; Sigma-Aldrich) as described [27]. Time-lapse experiments were made at 25, 28, 32 or 36°C, by acquiring epifluorescence cell images in single planes and 1×1 binning on an inverted microscope (Olympus IX71) equipped with a PlanApo 100x/1.40 IX70 objective and a Personal DeltaVision system (Applied Precision). Images were captured using CoolSnap HQ2 monochrome camera (Photometrics) and softWoRx 5.5.0 release 6 imaging software (Applied Precision). Subsequently, time-lapse CW, GFP and RFP images were restored and corrected by 3D Deconvolution (conservative ratio, 10 iterations and medium noise filtering) through softWoRx imaging software. Next, images were processed with Image J (National Institutes of Health) and Adobe Photoshop software. For time-lapses using maximum projection of SPB Cdc7-GFP and Cut11-GFP, images were obtained in z-stacks of 7 slices at 0.4 μm intervals. Then slices were processed with the function stacks and 3D projection of the Image J software.

Total fluorescence of Cdc7-GFP and Cdc13-GFP was quantified as described [29]. First, Image J software was used to correct the background fluorescence of each cell series through the Background subtraction from ROI function. Then, the sum of the remaining values of the pixels in the nucleus or SPB area was calculated. Similarly, total fluorescence of GFP-Etd1 in cell pole and middle was quantified as described in [18] by drawing the outlines of the tips and remaining medial region of the cell, and the sum of values of the pixels in each region was obtained with Image J software. Briefly, given that Etd1 spreads along the cell cortex in both poles and middle at the beginning of anaphase B, we followed the GFP-Etd1 intensity in these locations by drawing the corresponding ROIs of the cell poles and middle. The end of the ROI used to measure the cell tip continued with the end of the ROI used to measure the cell middle. Thus, the complete cell intensity was considered the sum of the three ROIs selected in the cell.

### Transmission electron microscopy

Early logarithmic phase wild-type cells were fixed with 2% glutaraldehyde EM (GA; Electron Microscopy Science) in 50 mM phosphate buffer pH 7.2, 150 mM NaCl (PBS) for 2 h at 4°C, post-fixed with 1.2% potassium permanganate overnight at 4°C and embedded in Quetol 812 as described [72–74]. Ultrathin sections were stained in 4% uranyl acetate and 0.4% lead citrate, and viewed with TEM H-800 (Hitachi) operating at 125 kV.

## Supporting information

S1 Fig The timing of septum deposition onset overlaps with chromosome mass separation from early anaphase B. **(A)** Early log-phase cells carrying GFP-Atb2 (tubulin), Hht1-RFP (histone H3) and RFP-Bgs1 were grown in YES at 28°C, stained with Calcofluor white (CW, 50 μg ml^-1^) and imaged by fluorescence microscopy. White arrow, first CW-stained septum synthesis detection. **(B, C, D)** Early log-phase cells carrying GFP-Bgs1 and Hht1-RFP (histone H3) (B), and Ags1-GFP with Hht1-RFP (C) or Nup107-GFP (nucleoporin, D) were grown and imaged by time-lapse microscopy as in Fig 1. Symbols are as in Fig 1. Anaphase B onset is considered as time zero (T = 0). **(E)** Septum synthesis always starts during the early stages of anaphase B in wild-type cells with different backgrounds. Table showing the time between the anaphase B onset and septation start (green), and between septation onset and the start of nuclear retraction (spindle disassembly, blue) in the indicated wild-type cells. Values are min ± SD. Values in parenthesis are the number of experiments (n) and analyzed cells, and the percentage of elapsed time of the corresponding period with respect to the total time required for the anaphase B process. Bars, 5 μm.
(TIF)

S2 Fig The presence of a non-degradable version of the Cdk1-associated cyclin Cdc13 causes cell cycle arrest in anaphase B and a blockage or delay in septation onset. **(A)** Cells expressing an endogenous non-degradable *cdc13*^*des2*^ version and a 45*-cdc13*^*+*^ wild-type copy, were grown at 28°C in the presence of thiamine for 10–15 h to repress the expression of the wild-type 45*-cdc13*^*+*^ copy, and imaged as in Fig 1. Values in parenthesis show the number of analyzed experiments and cells, and the percentage of cells in anaphase B with respect to the total cell number. Anaphase B onset is considered as time zero (T = 0). **(B)** Table showing the elapsed time between anaphase B onset and the start of septum synthesis (dark blue arrowhead) or the start of septum ingression (light blue arrowhead) in control wild-type cells and in cells as in A. Values are min ± SD and values in parenthesis are the increase in the timing of septation onset with respect to that of control wild-type cells 45-*cdc13*^+^ *cdc1*3^+^ (+T, 45-*cdc13*^*+*^ repressed). Arrowheads: dark blue, septum synthesis start; light blue, septum ingression onset. Other symbols are as in Fig 1. **(C)** The expression of an endogenous non-degradable Cdc13^des2^ version blocks the mitosis exit and restrains septation onset. Graph shows the percentages of cells in anaphase B (cells with two condensed chromosome masses) either with or without septum, in wild-type, 45*-cdc13*^*+*^ *cdc13*^*+*^, and 45*-cdc13*^*+*^ *cdc13*^*des2*^ strains. Cells were grown at 28°C either in the absence (-T, 45-*cdc13*^+^ induced) or in the presence of thiamine (+T, 45-*cdc13*^*+*^ repressed) for 15 h, and imaged by CW-staining and Hht1-RFP fluorescence microscopy. At least 190 cells of each strain and growth condition were examined. Error bars indicate standard deviation (SD). Bars, 5 μm.
(TIF)

S3 Fig The actomyosin ring is stably assembled in the cell middle before the recruitment of Bgs1 and septum synthesis start, and its function is required for the timely deposition of the septum in early anaphase B. **(A)** Timing of the major AR components and Sid2 stably localized as a ring to the division site with respect to Bgs1 ring localization and septation initiation in each analyzed case. For simplification, it is only shown the simultaneous kymographs of Sid2-GFP, RFP-Bgs1 and CW. Bgs1 and CW were also analyzed simultaneously with the rest of AR proteins and their localization is shown with the corresponding arrowhead. The cells of kymographs were grown and imaged as in Fig 1. Septation onset is considered as time zero (T = 0), which is the time immediately before the time of septum detection with CW. Arrowheads: green, AR and Sid2 proteins localization as a stable ring to the division site; red, Bgs1 localization as a stable ring to the division site; blue, CW-stained septum detection. The number of experiments (n) and cells analyzed in each case is shown. **(B)** Ags1 and Bgs4 stably localize to the division site (white arrow) after septation start, close before or after spindle disassembly (red arrowhead). Spindle disassembly is considered as time zero (T = 0). **(C)** Scheme showing the timing of localization as a stable ring in the division area of the proteins shown in A and B. Values are min ± SD. The number of experiments (n) and cells analyzed in each case is shown. **(D, E)** The timing of septation onset depends on the AR function. The start of septation is delayed when the functions of the AR unconventional type II myosin Myp2 (D) or the AR F-BAR protein Cdc15 (E) are compromised. The data of this figure are developed in Table 1 and Table 3. Growth conditions and symbols are as in Fig 1. Bars, 2 μm (A) and 5 μm (B, D, E).
(TIF)

S4 Fig Glucan synthases Bgs1, Bgs4 and Ags1 move to the division site during both the first and second rounds of mitosis after inactivation of the SIN signaling. **(A)** The displacement of the glucan synthases (GSs) to the division site does not depend on the SIN pathway. SIN *cdc11-119* mutant cells carrying Hht1-RFP and GFP-Bgs1 or GFP-Bgs4 were grown at 28°C, shifted to 36°C for 1 h to inactivate the SIN and imaged by time-lapse fluorescence microscopy (1 medial z slice, 6 or 10 min elapsed time). **(B)** Average time of the stable localization of the corresponding glucan synthase at the division site in the absence of SIN signaling. Time was quantified from cells either in the first or in the second mitotic round after SIN inactivation as depicted in A. GFP-Bgs1, n = 4, 24 cells; GFP-Bgs4, n = 3, 15 cells; and Ags1-GFP, n = 3, 13 cells. Error bars indicate standard deviation (SD). **(C)** SIN *cdc11-119* cells carrying AR Rlc1-RFP and GFP-Bgs4 were grown as in A and imaged by time-lapse fluorescence microscopy (1 medial z slice, 4 min elapsed time). White arrow: localization of Bgs4 to the cell middle after the transient formation of an unstable AR. Bars, 5 μm.
(TIF)

S5 Fig The establishment of SIN asymmetry and the timely activation of septum synthesis do not depend on each other. **(A, B)** Early log-phase wild-type and thermosensitive *cps1-191* (Bgs1) mutant cells were grown in YES at 25°C, shifted to 28°C for 1 h (A) or 32°C for 30 min (B) to produce a gradual delay in the onset of septum synthesis of *cps1-191* mutant, and imaged as in Fig 5C. Anaphase B onset is considered as time zero (T = 0). White arrow: first CW-stained detection of septum synthesis. Arrowheads: green, anaphase B onset; blue, septum deposition start (time immediately before septum detection with CW); red, complete asymmetry of SIN Cdc7, being Cdc7-GFP completely lost from one SPB. The data of this figure are developed in S2 Table. **(C)** The timing of septation onset is not related to the asymmetry of SIN. Early log-phase wild-type cells were grown in YES at 25°C, 28°C or 32°C, imaged as in Fig 5C and the timings of SIN asymmetry and of septation onset were determined with respect to the anaphase B onset (see also the data in S2 Table). Error bars indicate standard deviation (SD). Bars, 5 μm.
(TIF)

S6 Fig The activation of septum synthesis does not depend on the arrival of the SPB with active SIN Cdc7 to the cell tip vicinity. **(A, B)** The timing of septation activation depends on the SPB localization with respect to the cell middle. Cells with displaced SPBs present a delayed septation onset regardless of the location of the SPB containing the active SIN Cdc7. Early log-phase cells were grown and imaged as in Fig 5C (wild-type, A) or Fig 5D (*cdc25-*22, B). To displace the SPB to the cell tip, cells in early mitosis stage were centrifuged as in Fig 7 (3 min, 16000xg) and imaged. **(C)** The nucleus displacement does not alter the localization of Etd1 in the cell middle and division site where AR Rlc1 is localized. Early log-phase *etd1*^*+*^ 41X*-GFP-etd1*^*+*^ cells carrying Hht1-RFP and Rlc1-RFP were grown as in Fig 6A and either directly imaged (centered nucleus, left) or centrifuged to displace the nucleus as in A (right) and imaged by fluorescence microscopy. Symbols are as in Fig 1. Anaphase B onset is considered as time zero (T = 0). White arrow, band of Etd1 in the cell middle cortex. Bars, 5 μm.
(TIF)

S7 Fig Etd1 does not localize in the membrane of the incipient septum during anaphase B, instead it concentrates when the septum is advanced during telophase. Cells were grown in MM without thiamine (induced *GFP-etd1*^*+*^) at 32°C for 24 h and imaged as in Fig 1. Double-headed arrows, interval of septum formation without detectable Etd1 in the indicated cells; arrow, first detection of Etd1 along the septum membrane in telophase in the indicated cells. Bar, 5 μm.
(TIF)

S8 Fig The septum ingression onset in the absence of spindle MT coincides with the SPBs exit to the cytoplasmic face of the nuclear envelope. **(A)** Cells carrying the nuclear envelope nucleoporin Cut11-GFP either growing without MBC (upper panels) or with MBC as in Fig 7C (lower panels) were imaged by time-lapse fluorescence microscopy (maximum-intensity projections of 7 z slices at 0.4 μm intervals for Cut11-GFP and 1 medial z slice for CW-staining, 4 min elapsed time). Prophase onset was considered as time zero (T = 0). Arrowheads: black, SPBs entrance to the nuclear envelope (Cut11-GFP detected in the SPBs, prophase onset); pink, SPBs exit from the nuclear envelope (Cut11-GFP absent in the SPBs, anaphase onset); dark blue, septum synthesis start; light blue, septum ingression start. White arrowhead: first CW-stained septum synthesis detection. White arrow: first CW-staining increase showing septum ingression. **(B)** In the absence of spindle MT, Etd1 localizes to the centered or displaced division site where AR Rlc1 is located, independent of the nucleus position. Early log-phase *etd1*^*+*^ 41X*-GFP-etd1*^*+*^ cells carrying Hht1-RFP and Rlc1-RFP were grown as in Fig 6A and treated with MBC and centrifuged as in Fig 7 either to relocate nucleus, division site and Etd1 to a cell end or to separate the cell end-relocated nucleus from cell middle-remaining division site (AR Rlc1-RFP) and Etd1. Cells were imaged as in S6C Fig in the presence of MBC. White arrow, band of Etd1 in the cell middle cortex. Bars, 5 μm.
(TIF)

S1 Table Percentages of the elapsed time in each mitotic phase. (DOCX)

S2 Table The timing of septation onset is not related to the asymmetry of SIN. (DOCX)

S3 Table Fission yeast strains used in this study. (DOCX)

S4 Table Numerical data for graphs in Fig 1, Fig 2, Fig 3, Fig 4 and Fig 6. (PDF)
