# Supplementary figures and images for: Specific detection of fission yeast primary septum reveals septum and cleavage furrow ingression during early anaphase independent of mitosis completion
Source: PLoS Genet. 2018 May 29;14(5):e1007388. doi: 10.1371/journal.pgen.1007388 (PMC5993333; doi:10.1371/journal.pgen.1007388)

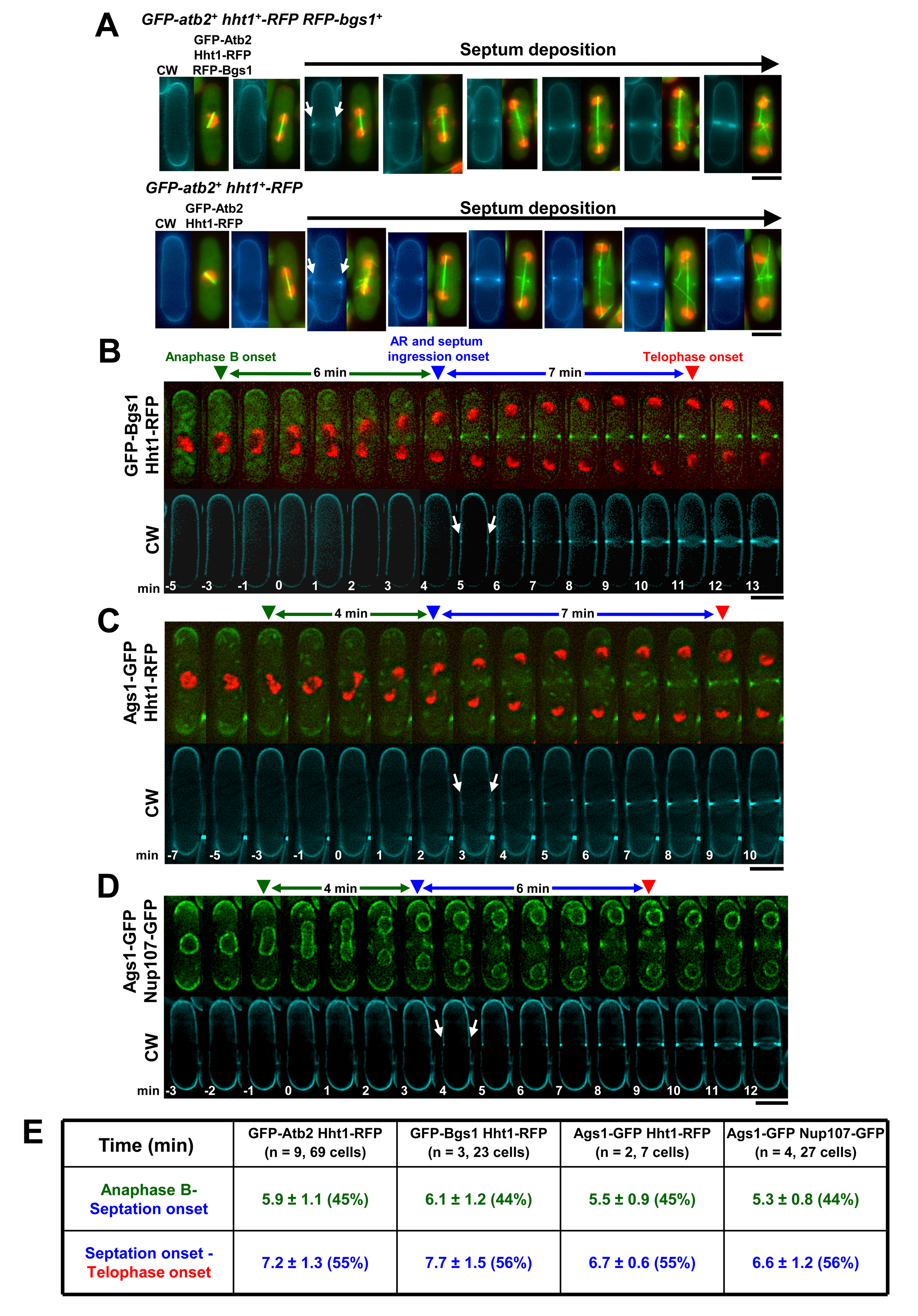

Supplement: S1 Fig — (A) Early log-phase cells carrying GFP-Atb2 (tubulin), Hht1-RFP (histone H3) and RFP-Bgs1 were grown in YES at 28°C, stained with Calcofluor white (CW, 50 μg ml-1) and imaged by fluorescence microscopy. White arrow, first CW-stained septum synthesis detection. (B, C, D) Early log-phase cells carrying GFP-Bgs1 and Hht1-RFP (histone H3) (B), and Ags1-GFP with Hht1-RFP (C) or Nup107-GFP (nucleoporin, D) were grown and imaged by time-lapse microscopy as in Fig 1. Symbols are as in Fig 1. Anaphase B onset is considered as time zero (T = 0). (E) Septum synthesis always starts during the early stages of anaphase B in wild-type cells with different backgrounds. Table showing the time between the anaphase B onset and septation start (green), and between septation onset and the start of nuclear retraction (spindle disassembly, blue) in the indicated wild-type cells. Values are min ± SD. Values in parenthesis are the number of experiments (n) and analyzed cells, and the percentage of elapsed time of the corresponding period with respect to the total time required for the anaphase B process. Bars, 5 μm. (TIF) [file pgen.1007388.s001.tif]

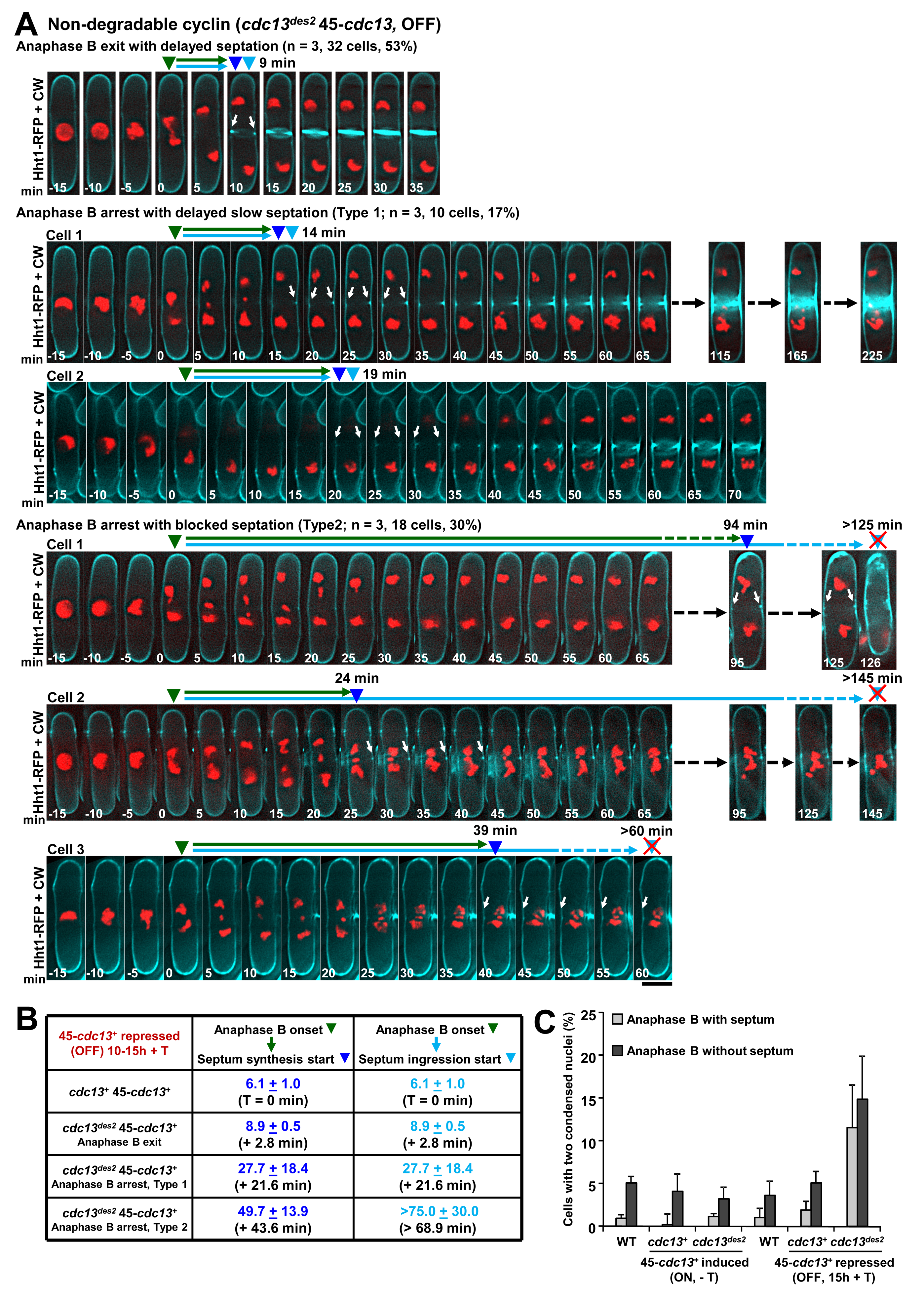

Supplement: S2 Fig — (A) Cells expressing an endogenous non-degradable cdc13des2 version and a 45-cdc13+ wild-type copy, were grown at 28°C in the presence of thiamine for 10–15 h to repress the expression of the wild-type 45-cdc13+ copy, and imaged as in Fig 1. Values in parenthesis show the number of analyzed experiments and cells, and the percentage of cells in anaphase B with respect to the total cell number. Anaphase B onset is considered as time zero (T = 0). (B) Table showing the elapsed time between anaphase B onset and the start of septum synthesis (dark blue arrowhead) or the start of septum ingression (light blue arrowhead) in control wild-type cells and in cells as in A. Values are min ± SD and values in parenthesis are the increase in the timing of septation onset with respect to that of control wild-type cells 45-cdc13+ cdc13+ (+T, 45-cdc13+ repressed). Arrowheads: dark blue, septum synthesis start; light blue, septum ingression onset. Other symbols are as in Fig 1. (C) The expression of an endogenous non-degradable Cdc13des2 version blocks the mitosis exit and restrains septation onset. Graph shows the percentages of cells in anaphase B (cells with two condensed chromosome masses) either with or without septum, in wild-type, 45-cdc13+ cdc13+, and 45-cdc13+ cdc13des2 strains. Cells were grown at 28°C either in the absence (-T, 45-cdc13+ induced) or in the presence of thiamine (+T, 45-cdc13+ repressed) for 15 h, and imaged by CW-staining and Hht1-RFP fluorescence microscopy. At least 190 cells of each strain and growth condition were examined. Error bars indicate standard deviation (SD). Bars, 5 μm. (TIF) [file pgen.1007388.s002.tif]

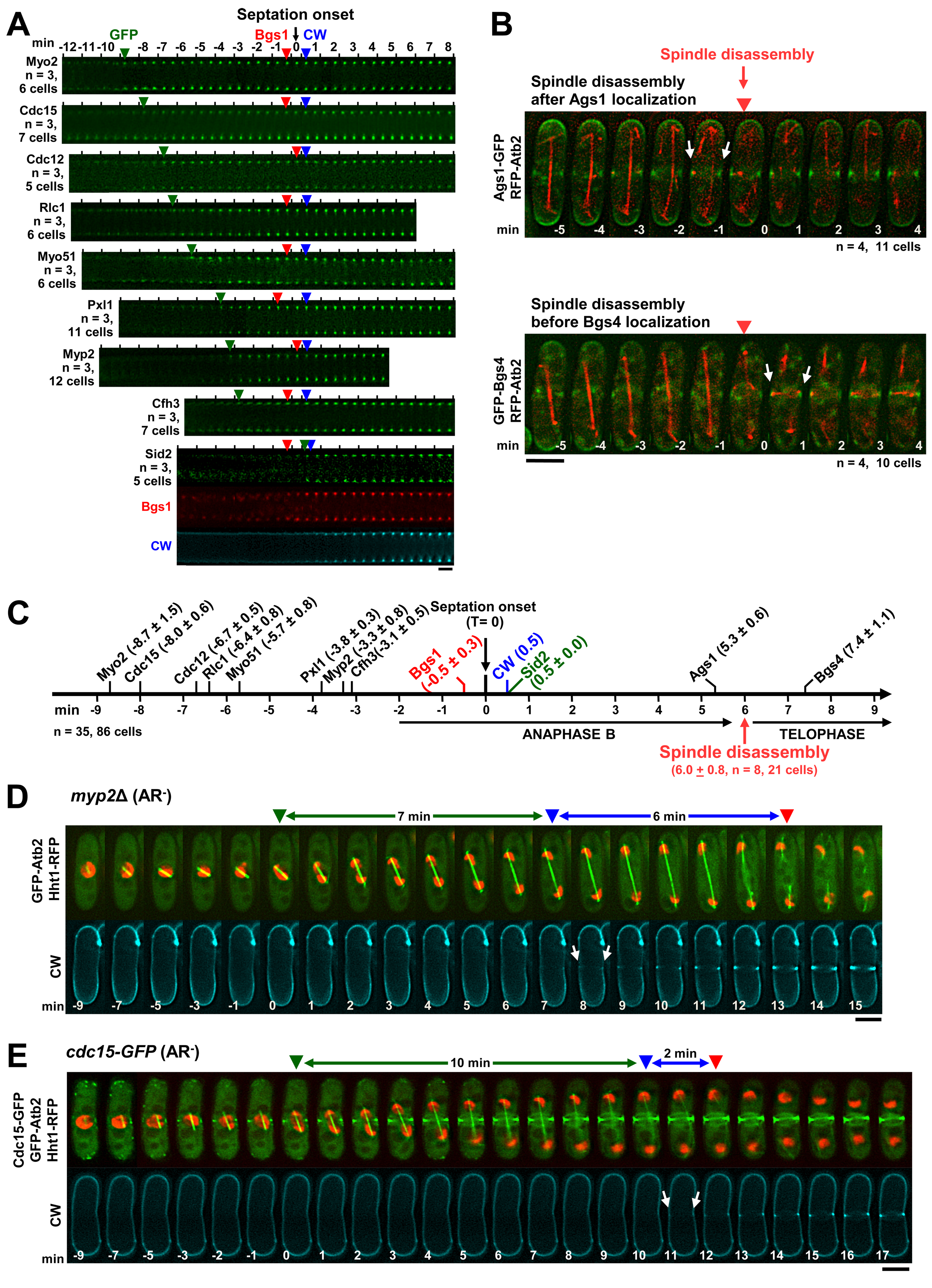

Supplement: S3 Fig — (A) Timing of the major AR components and Sid2 stably localized as a ring to the division site with respect to Bgs1 ring localization and septation initiation in each analyzed case. For simplification, it is only shown the simultaneous kymographs of Sid2-GFP, RFP-Bgs1 and CW. Bgs1 and CW were also analyzed simultaneously with the rest of AR proteins and their localization is shown with the corresponding arrowhead. The cells of kymographs were grown and imaged as in Fig 1. Septation onset is considered as time zero (T = 0), which is the time immediately before the time of septum detection with CW. Arrowheads: green, AR and Sid2 proteins localization as a stable ring to the division site; red, Bgs1 localization as a stable ring to the division site; blue, CW-stained septum detection. The number of experiments (n) and cells analyzed in each case is shown. (B) Ags1 and Bgs4 stably localize to the division site (white arrow) after septation start, close before or after spindle disassembly (red arrowhead). Spindle disassembly is considered as time zero (T = 0). (C) Scheme showing the timing of localization as a stable ring in the division area of the proteins shown in A and B. Values are min ± SD. The number of experiments (n) and cells analyzed in each case is shown. (D, E) The timing of septation onset depends on the AR function. The start of septation is delayed when the functions of the AR unconventional type II myosin Myp2 (D) or the AR F-BAR protein Cdc15 (E) are compromised. The data of this figure are developed in Table 1 and Table 3. Growth conditions and symbols are as in Fig 1. Bars, 2 μm (A) and 5 μm (B, D, E). (TIF) [file pgen.1007388.s003.tif]

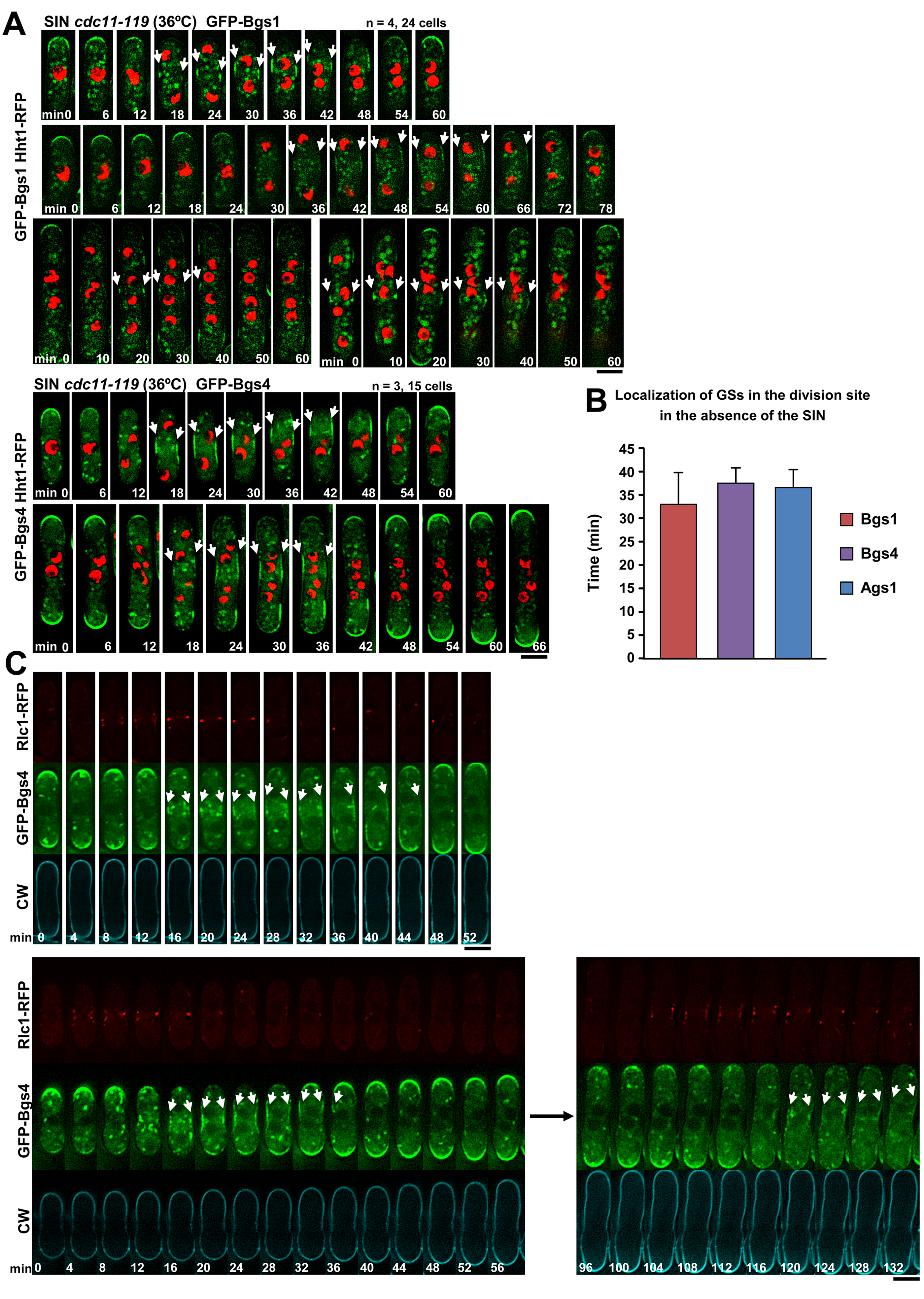

Supplement: S4 Fig — (A) The displacement of the glucan synthases (GSs) to the division site does not depend on the SIN pathway. SIN cdc11-119 mutant cells carrying Hht1-RFP and GFP-Bgs1 or GFP-Bgs4 were grown at 28°C, shifted to 36°C for 1 h to inactivate the SIN and imaged by time-lapse fluorescence microscopy (1 medial z slice, 6 or 10 min elapsed time). (B) Average time of the stable localization of the corresponding glucan synthase at the division site in the absence of SIN signaling. Time was quantified from cells either in the first or in the second mitotic round after SIN inactivation as depicted in A. GFP-Bgs1, n = 4, 24 cells; GFP-Bgs4, n = 3, 15 cells; and Ags1-GFP, n = 3, 13 cells. Error bars indicate standard deviation (SD). (C) SIN cdc11-119 cells carrying AR Rlc1-RFP and GFP-Bgs4 were grown as in A and imaged by time-lapse fluorescence microscopy (1 medial z slice, 4 min elapsed time). White arrow: localization of Bgs4 to the cell middle after the transient formation of an unstable AR. Bars, 5 μm. (TIF) [file pgen.1007388.s004.tif]

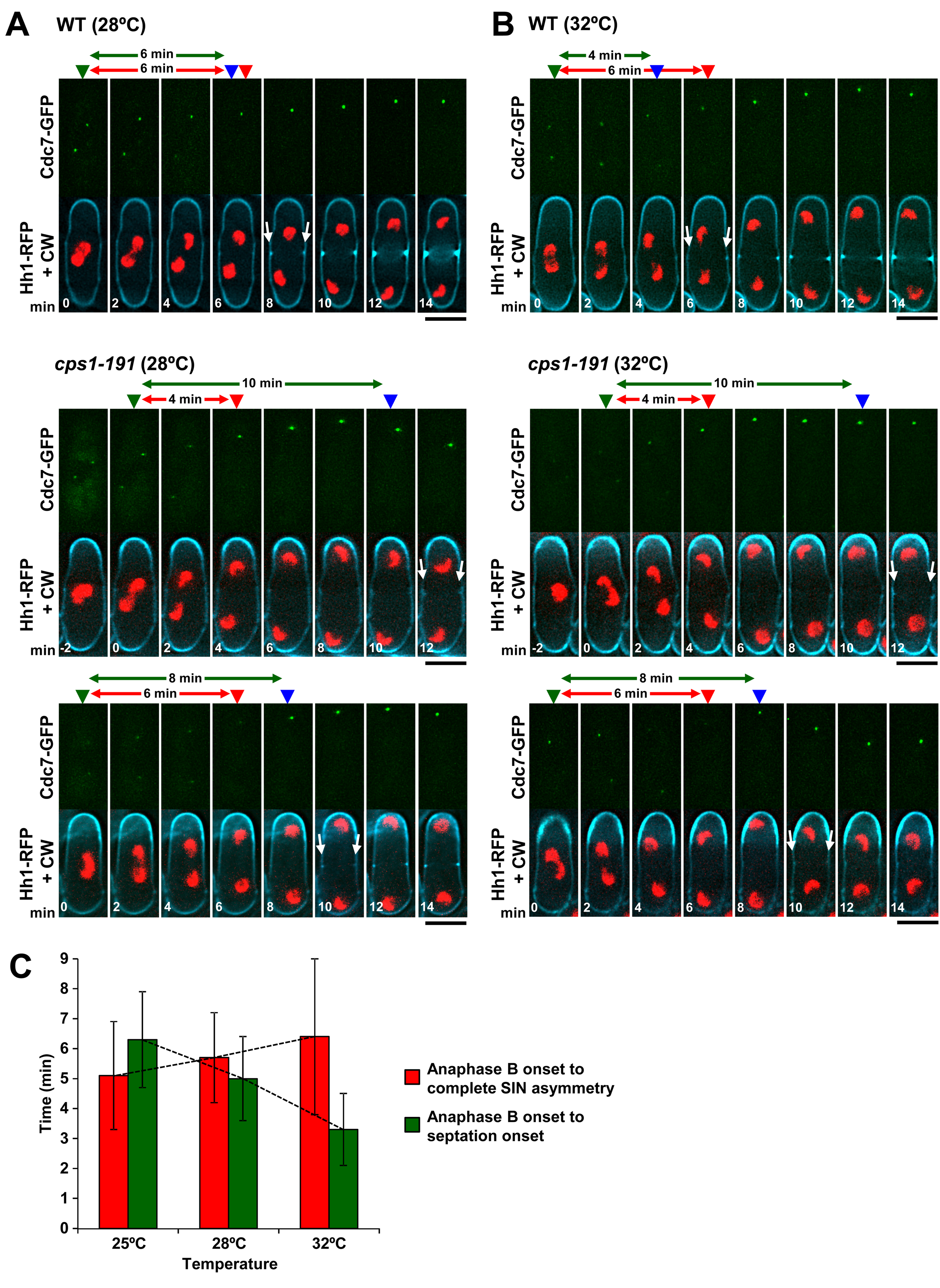

Supplement: S5 Fig — (A, B) Early log-phase wild-type and thermosensitive cps1-191 (Bgs1) mutant cells were grown in YES at 25°C, shifted to 28°C for 1 h (A) or 32°C for 30 min (B) to produce a gradual delay in the onset of septum synthesis of cps1-191 mutant, and imaged as in Fig 5C. Anaphase B onset is considered as time zero (T = 0). White arrow: first CW-stained detection of septum synthesis. Arrowheads: green, anaphase B onset; blue, septum deposition start (time immediately before septum detection with CW); red, complete asymmetry of SIN Cdc7, being Cdc7-GFP completely lost from one SPB. The data of this figure are developed in S2 Table. (C) The timing of septation onset is not related to the asymmetry of SIN. Early log-phase wild-type cells were grown in YES at 25°C, 28°C or 32°C, imaged as in Fig 5C and the timings of SIN asymmetry and of septation onset were determined with respect to the anaphase B onset (see also the data in S2 Table). Error bars indicate standard deviation (SD). Bars, 5 μm. (TIF) [file pgen.1007388.s005.tif]

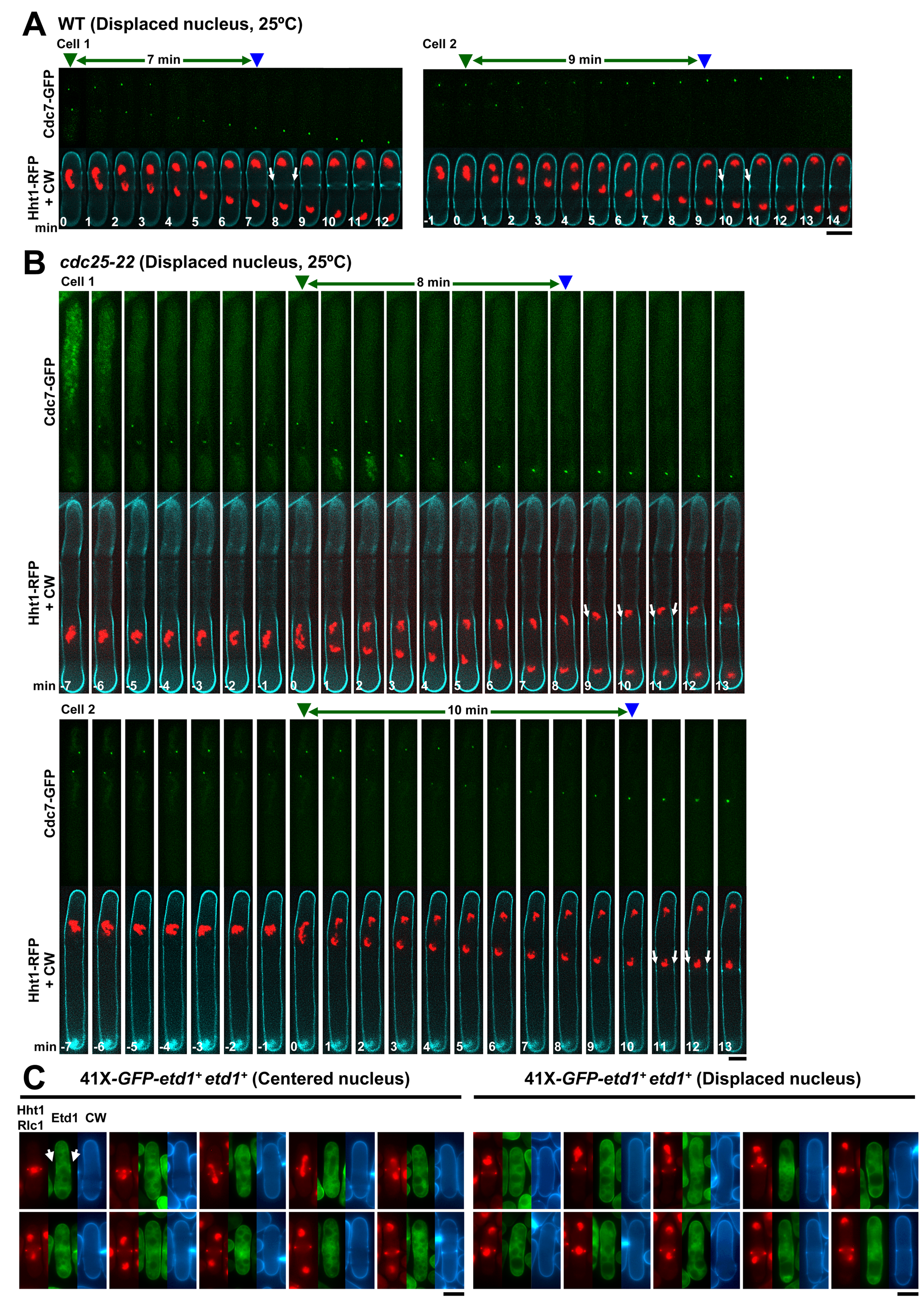

Supplement: S6 Fig — (A, B) The timing of septation activation depends on the SPB localization with respect to the cell middle. Cells with displaced SPBs present a delayed septation onset regardless of the location of the SPB containing the active SIN Cdc7. Early log-phase cells were grown and imaged as in Fig 5C (wild-type, A) or Fig 5D (cdc25-22, B). To displace the SPB to the cell tip, cells in early mitosis stage were centrifuged as in Fig 7 (3 min, 16000xg) and imaged. (C) The nucleus displacement does not alter the localization of Etd1 in the cell middle and division site where AR Rlc1 is localized. Early log-phase etd1+ 41X-GFP-etd1+ cells carrying Hht1-RFP and Rlc1-RFP were grown as in Fig 6A and either directly imaged (centered nucleus, left) or centrifuged to displace the nucleus as in A (right) and imaged by fluorescence microscopy. Symbols are as in Fig 1. Anaphase B onset is considered as time zero (T = 0). White arrow, band of Etd1 in the cell middle cortex. Bars, 5 μm. (TIF) [file pgen.1007388.s006.tif]

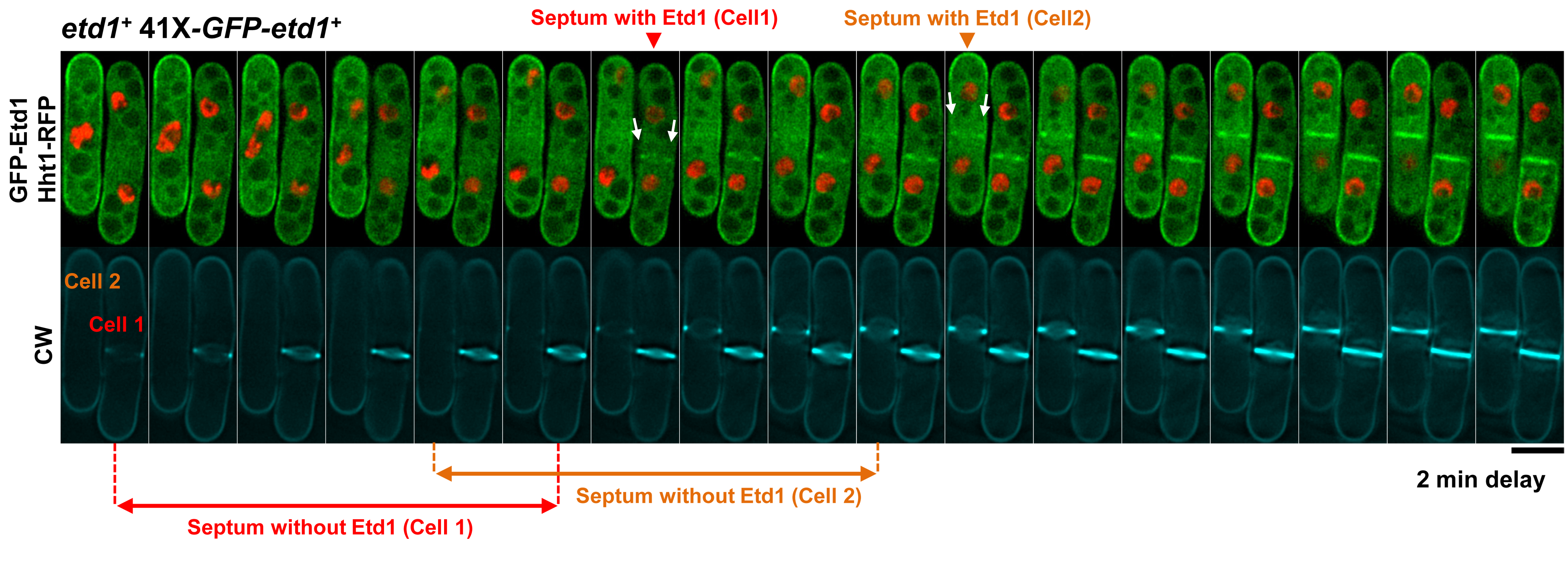

Supplement: S7 Fig — Cells were grown in MM without thiamine (induced GFP-etd1+) at 32°C for 24 h and imaged as in Fig 1. Double-headed arrows, interval of septum formation without detectable Etd1 in the indicated cells; arrow, first detection of Etd1 along the septum membrane in telophase in the indicated cells. Bar, 5 μm. (TIF) [file pgen.1007388.s007.tif]

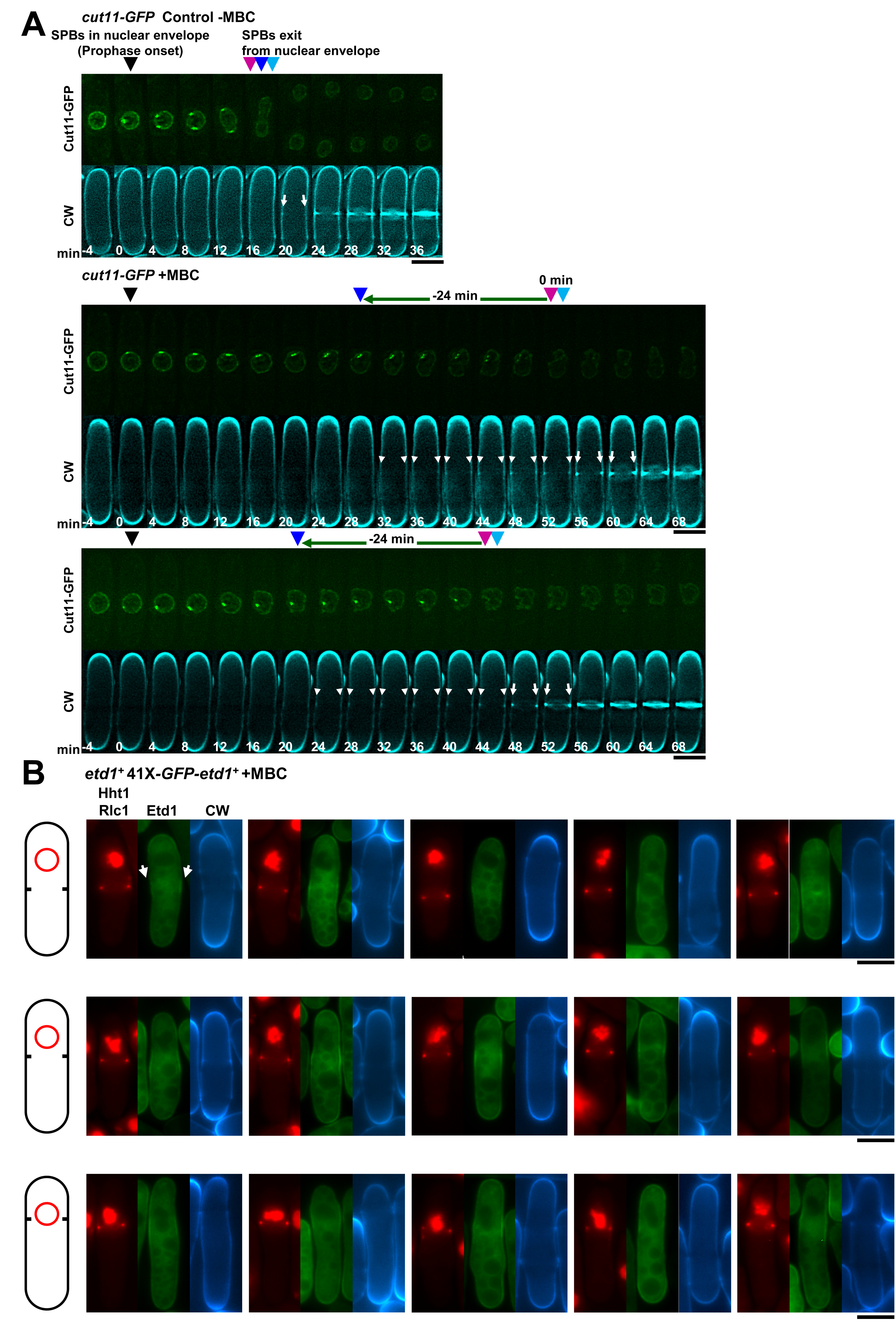

Supplement: S8 Fig — (A) Cells carrying the nuclear envelope nucleoporin Cut11-GFP either growing without MBC (upper panels) or with MBC as in Fig 7C (lower panels) were imaged by time-lapse fluorescence microscopy (maximum-intensity projections of 7 z slices at 0.4 μm intervals for Cut11-GFP and 1 medial z slice for CW-staining, 4 min elapsed time). Prophase onset was considered as time zero (T = 0). Arrowheads: black, SPBs entrance to the nuclear envelope (Cut11-GFP detected in the SPBs, prophase onset); pink, SPBs exit from the nuclear envelope (Cut11-GFP absent in the SPBs, anaphase onset); dark blue, septum synthesis start; light blue, septum ingression start. White arrowhead: first CW-stained septum synthesis detection. White arrow: first CW-staining increase showing septum ingression. (B) In the absence of spindle MT, Etd1 localizes to the centered or displaced division site where AR Rlc1 is located, independent of the nucleus position. Early log-phase etd1+ 41X-GFP-etd1+ cells carrying Hht1-RFP and Rlc1-RFP were grown as in Fig 6A and treated with MBC and centrifuged as in Fig 7 either to relocate nucleus, division site and Etd1 to a cell end or to separate the cell end-relocated nucleus from cell middle-remaining division site (AR Rlc1-RFP) and Etd1. Cells were imaged as in S6C Fig in the presence of MBC. White arrow, band of Etd1 in the cell middle cortex. Bars, 5 μm. (TIF) [file pgen.1007388.s008.tif]
