# Supplementary material for: Specific detection of fission yeast primary septum reveals septum and cleavage furrow ingression during early anaphase independent of mitosis completion
Source: PLoS Genet. 2018 May 29;14(5):e1007388. doi: 10.1371/journal.pgen.1007388 (PMC5993333; doi:10.1371/journal.pgen.1007388)
Supplement: S1 Table — (DOCX) [file pgen.1007388.s009.docx]

| **S1 Table. Percentages of the elapsed time in each mitotic phase.** | | | |
| --- | --- | --- | --- |
| **Strains** | **Prophase/Metaphase/Anaphase A^1^** | **Anaphase B** | |
| **25ºC** |  | Septation onset^2^ | Spindle disassembly^3^ |
| **Wild-type** (51 cells) | 47.6 + 3.3% | 24.8 + 4.8% | 27.6 + 7.3% |
| ***wee1-50*** (15 cells) | 45.0 + 5.2% | 19.4 + 4.5% | 35.6 + 8.1% |
| ***cdc2-3W*** (16 cells) | 48.3 + 4.7% | 21.2 + 5.5% | 30.5 + 6.2% |
| ***cdc10-119*** (11 cells) | 41.9 + 4.7% | 20.0 + 2.1% | 38.1 + 7.4% |
| ***cdc25-22*** (32 cells) | 42.2 + 4.6% | 22.7 + 4.2% | 35.1 + 3.3% |
| **28ºC** | | | |
| **Wild-type** (126 cells) | 49.2 + 3.4% | 22.6 + 4.0% | 28.2 + 4.4% |
| ***cdc13^+^-GFP*** (20 cells) | 51.8 + 2.0% | 25.2 + 3.5% | 23.0 + 5.3% |
| ***cdc15-GFP*** (14 cells) | 46.3 + 4.4% | 41.2 + 5.1% | 12.5 + 4.8% |
| ***myp2*Δ** (10 cells) | 49.6 + 4.1% | 27.6 + 3.0% | 22.8 + 6.0% |
| Values are percentage of total mitosis time ± SD from the time intervals shown in Table 1 (28ºC) and Table 2 (25ºC).  ^1^ Percentage of elapsed time from the start of prophase to the end of anaphase A with respect to the total mitosis time.  ^2^ Percentage of elapsed time from the start of anaphase B to the onset of septation with respect to the total mitosis time.  ^3^ Percentage of elapsed time from the onset of septation to the spindle disassembly with respect to the total mitosis time. | | | |
