# Supplementary material for: Specific detection of fission yeast primary septum reveals septum and cleavage furrow ingression during early anaphase independent of mitosis completion
Source: PLoS Genet. 2018 May 29;14(5):e1007388. doi: 10.1371/journal.pgen.1007388 (PMC5993333; doi:10.1371/journal.pgen.1007388)
Supplement: S2 Table — (DOCX) [file pgen.1007388.s010.docx]

| **S2 Table. The timing of septation onset is not related to the asymmetry of SIN.** | | | | |
| --- | --- | --- | --- | --- |
| **Temperature** | **Strain** | **Anaphase B onset**  **to complete SIN asymmetry^1^** | **Anaphase B onset**  **to septation onset^2^** | **SIN asymmetry to septation onset^3^** |
| 25ºC | *cdc7^+^-GFP hht1^+^-RFP* (n=3, 23 cells)^4^ | 5.1 + 1.8 | 6.3 + 1.6 | + 1.2 |
| 28ºC | *cdc7^+^-GFP hht1^+^-RFP* (n=3, 23 cells) | 5.7 + 1.5 | 5.0 + 1.4 | - 0.7 |
| 32ºC | *cdc7^+^-GFP hht1^+^-RFP* (n=2, 19 cells) | 6.4 + 2.6 | 3.3 + 1.2 | - 3.1 |
| 25ºC | *cdc7^+^-GFP hht1^+^-RFP csc2*Δ (n=2, 13 cells) | > 33.1 + 6.3 | 6.3 + 1.2 | > - 26.8 |
| 25ºC | *cdc7^+^-GFP hht1^+^-RFP cdc25-22* (n=2, 12 cells) | 9.1 + 2.2 | 10.7 + 2.2 | + 1.6 |
| 28ºC | *cdc7^+^-GFP hht1^+^-RFP cps1-191* (n=3, 20 cells) | 6.3 + 2.0 | 9.1 + 1.5 | + 2.8 |
| 32ºC | *cdc7^+^-GFP hht1^+^-RFP cps1-191* (n=2, 20 cells) | 5.9 + 1.5 | 11.9 + 4.2 | + 6.0 |
| Values are minutes + SD.  ^1^ Time between the anaphase B start and the complete SIN asymmetry.  ^2^ Time between the anaphase B start and the septation onset.  ^3^ Time between SIN asymmetry and septation onset.  ^4^ The value n is the number of experiments in each case. | | | | |
