# Supplementary material for: Specific detection of fission yeast primary septum reveals septum and cleavage furrow ingression during early anaphase independent of mitosis completion
Source: PLoS Genet. 2018 May 29;14(5):e1007388. doi: 10.1371/journal.pgen.1007388 (PMC5993333; doi:10.1371/journal.pgen.1007388)
Supplement: S3 Table — (DOCX) [file pgen.1007388.s011.docx]

| **S3 Table. Fission yeast strains used in this study.** | | |
| --- | --- | --- |
| **Strain** | **Genotype** | **Source** |
| 33 | 972 h^-^ | P. Munz^a^ |
| 37 | 975 h^+^ | P. Munz^a^ |
| 251 | *leu1-32 ura4-*Δ*18 his3-*Δ*1* *ade6-M210* h^+^ | J. Ribas |
| 274 | *leu1-32 ura4-*Δ*18 his3-*Δ*1* *ade6-M210* h^-^ | J. Ribas |
| 284 | *leu1-32 ura4-*Δ*18 his3-*Δ*1* h^-^ | J. Ribas |
| 285 | *leu1-32 ura4-*Δ*18 his3-*Δ*1* h^+^ | J. Ribas |
| 419 | *leu1-32 ura4-*Δ*18* h^-^ | J. Ribas |
| 420 | *leu1-32 ura4-*Δ*18* h^+^ | J. Ribas |
| 458 | *ura4-*Δ*18 lys1-131* h^+^ | J. Ribas |
|  | | |
| 461 | *leu1-32 cdc10-129* h^-^ | P. Nurse^b^ |
| 472 | *leu1-32 ura4-*Δ*18 cdc10-129* h^-^ | This study |
| 462 | *leu1-32 cdc25-22* h^+^ | P. Nurse^b^ |
| 476 | *leu1-32 ura4-*Δ*18 cdc25-22* h^-^ | This study |
| 5305 | *ura4-*Δ*18 wee1-50* h^-^ | P. Nurse^b^ |
| 5434 | *cdc2-3W:NatMX6* h^-^ | P. Nurse^b^ |
| 899 | *leu1-32 his3-*Δ*1 cdc11-119* h^+^ | P. Nurse^b^ |
| 2326 | *leu1-32 ura4-*Δ*18 ade6-M216 sid2-250* h^+^ | V. Simanis^c^ |
| 5680 | *leu1-32 ura4-*Δ*18 ade6-M210 csc2*Δ*::KanMX6* h^+^ | M. Balasubramanian^d^ |
| 5701 | *ura4-*Δ*18 csc2*Δ*::KanMX6* h^-^ | This study |
| 1024 | *leu1-32 ura4-*Δ*18 cps1-191* h^-^ | M. Balasubramanian^d^ |
| 5425 | *leu1-32 ura4-*Δ*18 ade6-M210 mad2*Δ*::KanMX6* h^+^ | S. Moreno |
| 1105 | *leu1-32 ura4-*Δ*18 ade6-M216 myp2*Δ*::ura4^+^* h^+^ | I. Mabuchi^e^ |
| 1149 | *leu1-32 ura4-*Δ*18 his3-*Δ*1 ade6-M210 myp2*Δ*::ura4^+^* h^-^ | This study |
| 6095 | *cdc2-asM17::NatMX6* h^-^ | S. Moreno |
|  | | |
| 5727 | *leu1-32 ura4-*Δ*18 ade6-704 cdc13^+^:leu1^+^:*P*nmt1^+^-*45*-cdc13^+^:sup3-5 cdc13*Δ*::ura4^+^* h^-^/h^-^ | S. Moreno |
| 5728 | *leu1-32 ura4-*Δ*18 ade6-704 cdc13^des2^:leu1^+^* P*nmt1^+^-*45*-cdc13^+^:sup3-5 cdc13*Δ*::ura4^+^* h^-^/h^-^ | S. Moreno |
| 6100 | *leu1-32 ura4-*Δ*18 ade6-M216 etd1∆::ura4*^+^ P*nmt1^+^*-41X-*GFP*-*etd1*Δ*1-400:leu1^+^* h^+^ | J. Jiménez^n^ |
|  | | |
| 3915 | *ura4-*Δ*18 his3-*Δ*1 nup107^+^-GFP:KanMX6* h^+^ | F. Chang^f^ |
| 4047 | *leu1-32 ura4-*Δ*18 his3-*Δ*1 ade6-M210 hht1^+^-RFP:KanMX6* h^-^ | J. Cooper^g^ |
| 5653 | *leu1-32 ura4-*Δ*18 hht1^+^-RFP:KanMX6* h^+^ | This study |
| 5654 | *leu1-32 ura4-*Δ*18 hht1^+^-RFP:KanMX6* h^-^ | This study |
| 5657 | *leu1-32 ura4-*Δ*18 his3-*Δ*1 ade6-M210 hht1^+^-RFP:KanMX6* h^+^ | This study |
| 982 | *leu1-32 ura4-*Δ*18 cdc15^+^-GFP:ura4^+^* h^-^ | S. Moreno |
| 1350 | *leu1-32 ura4-*Δ*18 myp2^+^-GFP:ura4^+^* h^-^ | M. Balasubramanian^d^ |
| 1755 | *leu1-32 ura4-*Δ*18 rlc1^+^-GFP:KanMX6* h^+^ | V. Simanis^c^ |
| 4868 | *leu1-32 ura4-*Δ*18 rlc1^+^-tdTom:NatMX6* h^-^ | P. Pérez |
| 4869 | *leu1-32 ura4-*Δ*18 rlc1^+^-tdTom:NatMX6* h^+^ | P. Pérez |
| 1337 | *leu1-32 ura4-*Δ*18 ade6-M210 myp2^+^-GFP**:KanMX6* h^-^ | T. Pollard^h^ |
| 1757 | *leu1-32 ura4-*Δ*18 ade6-M210 GFP-cdc15^+^:KanMX6* h^-^ | T. Pollard^h^ |
| 1777 | *leu1-32 ura4-*Δ*18 ade6-M210 GFP-cdc15^+^:KanMX6* h^+^ | This study |
| 3325 | *leu1-32 ura4-*Δ*18 myo51^+^-GFP:KanMX6* h^+^ | J. Hyams^i^ |
| 3538 | *leu1-32 ura4-*Δ*18 pxl1*Δ*::KanMX6 GFP-pxl1^+^:leu1^+^* h^+^ | P. Pérez |
| 3543 | *leu1-32 ura4-*Δ*18 his3-*Δ*1 ade6-M210 cfh3*Δ:*:KanMX6 GFP-cfh3^+^:leu1^+^* h^+^ | H. Valdivieso^j^ |
| 2525 | *ura4-*Δ*18 leu1::GFP-atb2^+^:ura4^+^* h^+^ | V. Simanis^c^ |
| 5044 | *leu1-32 ura4-*Δ*18 Cher-atb2^+^:AuR* h^-^ | T. Toda^k^ |
| 2848 | *leu1-32 ura4-*Δ*18 sid2^+^-GFP:ura4^+^* h^+^ | V. Simanis^c^ |
| 3596 | *leu1-32 ura4-*Δ*18 cdc7^+^-GFP:ura4^+^* h^+^ | D. McCollum^l^ |
| 6094 | *leu1-32 mob1^+^-GFP:KanMX6* h^-^ | J. Jiménez^n^ |
|  | | |
| 1723 | *leu1-32 ura4-*Δ*18 his3-*Δ*1 bgs1*Δ*::ura4^+^* P*bgs1^+^::GFP-12A-bgs1^+^:leu1^+^* h^+^ | J. Ribas |
| 1781 | *leu1-32 ura4-*Δ*18 his3-*Δ*1 bgs1*Δ*::ura4^+^* P*bgs1^+^::tdTom-12A-bgs1^+^:leu1^+^* h^-^ | J. Ribas |
| 3591 | *leu1-32 ura4-*Δ*18 his3-*Δ*1 bgs1*Δ*::ura4^+^* P*bgs1^+^::tdTom-12A-bgs1^+^:leu1^+^* h^+^ | J. Ribas |
| 2285 | *leu1-32 ura4-*Δ*18 his3-*Δ*1 bgs4*Δ*::ura4^+^* P*bgs4^+^::2xGFP-12A-bgs4^+^:leu1^+^* h^+^ | J. Ribas |
| 2365 | *leu1-32 ura4-*Δ*18 his3-*Δ*1 bgs4*Δ*::ura4^+^* P*bgs4^+^::GFP-12A-bgs4^+^:leu1^+^* h^+^ | J. Ribas |
| 3166 | *leu1-32 ura4-*Δ*18 his3-*Δ*1 ade6-M210 ags1*Δ 3’UTR*_ags1+_::ags1^+^-12A-GFP-12A:leu1^+^:ura4*^+^ h^-^ | J. Ribas |
| 3167 | *leu1-32 ura4-*Δ*18 his3-*Δ*1 ade6-M210 ags1*Δ 3’UTR*_ags1+_::ags1^+^-12A-GFP-12A:leu1^+^:ura4*^+^ h^+^ | J. Ribas |
| 3168 | *leu1-32 ura4-*Δ*18 his3-*Δ*1 ags1*Δ 3’UTR*_ags1+_::ags1^+^-12A-GFP-12A:leu1^+^:ura4*^+^ h^-^ | J. Ribas |
| **Used for experiments in Fig 1, Fig 2, Fig 3, Fig 4, Fig 8, and S1 Fig** | | |
| 4158 | *ura4-*Δ*18 leu1::GFP-atb2^+^:ura4^+^ hht1^+^-RFP:KanMX6* h^+^ | This study |
| 5431 | *ura4-*Δ*18 leu1::GFP-atb2^+^:ura4^+^ hht1^+^-RFP:KanMX6* h^-^ | This study |
| 5526 | *ura4-*Δ*18 leu1::GFP-atb2^+^:ura4^+^ hht1^+^-RFP:KanMX6 bgs1*Δ*::ura4^+^* P*bgs1^+^::tdTom-12A-bgs1^+^:leu1^+^* h^-^ | This study |
| 4078 | *leu1-32 ura4-*Δ*18 his3-*Δ*1 ade6-M210 hht1^+^-RFP:KanMX6 bgs1*Δ*::ura4^+^* P*bgs1^+^::GFP-12A-bgs1^+^:leu1^+^* h^+^ | This study |
| 3979 | *leu1-32 ura4-*Δ*18 his3-*Δ*1 ade6-M210 nup107^+^-GFP:KanMX6 ags1*Δ  3’UTR*_ags1+_::ags1^+^-12A-GFP-12A:leu1^+^:ura4*^+^ h^-^ | This study |
| 4082 | *leu1-32 ura4-*Δ*18 his3-*Δ*1 ade6-M210 hht1^+^-RFP:KanMX6* *ags1*Δ 3’UTR*_ags1+_::ags1^+^-12A-GFP-12A:leu1^+^:ura4*^+^ h^+^ | This study |
| 5455 | *cdc13^+^-GFP:KanMX6* h^-^ | P. Nurse^b^ |
| 5700 | *cdc13^+^-GFP:KanMX6* h^+^ | This study |
| **Used for experiments in Fig 2 and Fig 3** | | |
| 5625 | *leu1^+^/ leu1::GFP-atb2^+^:ura4^+^ ura4-*Δ*18/ura4-*Δ*18 lys1-131/lys1^+^* *hht1^+^-RFP:KanMX6/hht1^+^* h^+^/ h^-^ | This study |
| 5631 | *leu1-32/leu1^+^ ura4-*Δ*18/ura4-*Δ*18 his3-*Δ*1/his3^+^ ade6-M210/ade6^+^ lys1-131/lys1^+^ hht1^+^-RFP:KanMX6/hht1^+^* h^+^/ h^-^ | This study |
| 5398 | *ura4-*Δ*18 cdc10-129* *leu1::GFP-atb2^+^:ura4^+^ hht1^+^-RFP:KanMX6* h^-^ | This study |
| 5400 | *ura4-*Δ*18 cdc25-22* *leu1::GFP-atb2^+^:ura4^+^ hht1^+^-RFP:KanMX6* h^+^ | This study |
| 5428 | *ura4-*Δ*18 wee1-50 leu1::GFP-atb2^+^:ura4^+^ hht1^+^-RFP:KanMX6* h^+^ | This study |
| 5450 | *ura4-*Δ*18 cdc2-3W:NatMX6 leu1::GFP-atb2^+^:ura4^+^ hht1^+^-RFP:KanMX6* h^+^ | This study |
| **Used for experiments in Fig 4, Fig 7, Fig 8, S2 Fig and S7 Fig** | | |
| 5426 | *leu1-32 ura4-*Δ*18 cdc13-117:cdc13^+^-GFP:LEU2* h^-^ | M. Yanagida^m^ |
| 5478 | *cdc25-22 cdc13^+^-GFP:KanMX6* h^+^ | This study |
| 5735 | *leu1-32 ura4-*Δ*18 hht1^+^-RFP:KanMX6 cdc13^+^:leu1^+^* P*nmt1^+^-*45*-cdc13^+^:sup3-5 cdc13*Δ*::ura4^+^* h^-^/h^-^ | This study |
| 5736 | *leu1-32 ura4-*Δ*18 hht1^+^-RFP:KanMX6 cdc13^des2^:leu1^+^* P*nmt1^+^-*45*-cdc13^+^:sup3-5 cdc13*Δ*::ura4^+^* h^-^/h^-^ | This study |
| 5763 | *leu1-32 cdc13^+^-GFP:KanMX6 mad2*Δ*::KanMX6* h^+^ | This study |
| 5904 | *cdc13^+^-GFP:KanMX6* *sid2-250* h^-^ | This study |
| 6096 | *cut11^+^-GFP:ura4^+^* *leu1-32* h^+^ | P. Pérez |
| 6119 | *cdc13^+^-GFP:KanMX6*  *cdc2-asM17::NatMX6* h^+^ | This study |
| **Used for experiments in S3 Fig and S4 Fig** | | |
| 3309 | *leu1-32 ura4-*Δ*18 cdc12^+^-3xGFP:KanMX6 bgs1*Δ*::ura4^+^* P*bgs1^+^::tdTom-12A-bgs1^+^:leu1^+^* h^+^ | This study |
| 3311 | *leu1-32 ura4-*Δ*18 his3-*Δ*1 rlc1^+^-GFP:KanMX6 bgs1*Δ*::ura4^+^* P*bgs1^+^::tdTom-12A-bgs1^+^:leu1^+^* h^-^ | This study |
| 3340 | *leu1-32 ura4-*Δ*18 his3-*Δ*1 myo51^+^-GFP:KanMX6 bgs1*Δ*::ura4^+^* P*bgs1^+^::tdTom-12A-bgs1^+^:leu1^+^* h^+^ | This study |
| 3732 | *leu1-32 ura4-*Δ*18 myp2^+^-GFP:KanMX6 bgs1*Δ*::ura4^+^* P*bgs1^+^::tdTom-12A-bgs1^+^:leu1^+^* h^+^ | This study |
| 3734 | *leu1-32 ura4-*Δ*18 myo2^+^-GFP:ura4^+^ bgs1*Δ*::ura4^+^* P*bgs1^+^::tdTom-12A-bgs1^+^:leu1^+^* h^+^ | This study |
| 3755 | *leu1-32 ura4-*Δ*18 his3-*Δ*1 ade6-M210 cfh3*Δ:*:KanMX6 GFP-cfh3^+^:leu1^+^ bgs1*Δ*::ura4^+^*  P*bgs1^+^::tdTom-12A-bgs1^+^:leu1^+^* h^+^ | This study |
| 4306 | *leu1-32 ura4-*Δ*18 his3-*Δ*1 GFP-cdc15^+^:KanMX6 bgs1*Δ*::ura4^+^* P*bgs1^+^::tdTom-12A-bgs1^+^:leu1^+^* h^+^ | This study |
| 4326 | *leu1-32 ura4-*Δ*18 pxl1*Δ*::KanMX6 GFP-pxl1^+^:leu1^+^ bgs1*Δ*::ura4^+^* P*bgs1^+^::tdTom-12A-bgs1^+^:leu1^+^* h^+^ | This study |
| 5097 | *leu1-32 ura4-*Δ*18 Cher-atb2^+^:AuR bgs4*Δ*::ura4^+^* P*bgs4^+^::GFP-12A-bgs4^+^:leu1^+^* h^-^ | This study |
| 5095 | *leu1-32 ura4-*Δ*18 his3-*Δ*1 Cher-atb2^+^:AuR ags1*Δ 3’UTR*_ags1+_::ags1^+^-12A-GFP-12A:leu1^+^:ura4*^+^ h^+^ | This study |
| 4084 | *leu1-32 ura4-*Δ*18 his3-*Δ*1 ade6-M210 cdc11-119 hht1^+^-RFP:KanMX6* h^-^ | This study |
| 4085 | *leu1-32 ura4-*Δ*18 his3-*Δ*1 ade6-M210 cdc11-119 hht1^+^-RFP:KanMX6* h^+^ | This study |
| 4081 | *leu1-32 ura4-*Δ*18 his3-*Δ*1 ade6-M210 hht1^+^-RFP:KanMX6 bgs4*Δ*::ura4^+^* P*bgs4^+^::GFP-12A-bgs4^+^:leu1^+^* h^+^ | This study |
| 4121 | *leu1-32 ura4-*Δ*18 his3-*Δ*1 ade6-M210 cdc11-119 hht1^+^-RFP:KanMX6 bgs1*Δ*::ura4^+^*  P*bgs1^+^::GFP-12A-bgs1^+^:leu1^+^* h^+^ | This study |
| 4123 | *leu1-32 ura4-*Δ*18 his3-*Δ*1 ade6-M210 cdc11-119 hht1^+^-RFP:KanMX6 bgs4*Δ*::ura4^+^*  P*bgs4^+^::GFP-12A-bgs4^+^:leu1^+^* h^+^ | This study |
| 6121 | *leu1-32 ura4-*Δ*18 his3-*Δ*1 cdc11-119 rlc1^+^-tdTom:KanMX6 bgs4*Δ*::ura4^+^*  P*bgs4^+^::GFP-12A-bgs4^+^:leu1^+^* h^+^ | This study |
| 5402 | *ura4-*Δ*18 cdc15^+^-GFP:ura4^+^ leu1::GFP-atb2^+^:ura4^+^ hht1^+^-RFP:KanMX6* h^+^ | This study |
| 5444 | *ura4-*Δ*18 his3-*Δ*1 myp2*Δ*::ura4^+^ leu1::GFP-atb2^+^:ura4^+^ hht1^+^-RFP:KanMX6* h^+^ | This study |
| **Used for experiments in Fig 5, S3 Fig, S5 Fig and S6 Fig** | | |
| 3326 | *leu1-32 ura4-*Δ*18 sid2^+^-GFP:ura4^+^ bgs1*Δ*::ura4^+^* P*bgs1^+^::tdTom-12A-bgs1^+^:leu1^+^* h^-^ | This study |
| 6133 | *leu1-32 ura4-*Δ*18 his3-*Δ*1 mob1^+^-GFP:KanMX6*  *bgs1*Δ*::ura4^+^* P*bgs1^+^::tdTom-12A-bgs1^+^:leu1^+^* h^+^ | This study |
| 4014 | *leu1-32 ura4-*Δ*18 ade6-M210 sid2-250 ags1*Δ 3’UTR*_ags1+_::ags1^+^-12A-GFP-12A:leu1^+^:ura4*^+^ h^+^ | This study |
| 4083 | *leu1-32 ura4-*Δ*18 his3-*Δ*1 ade6-M210 ags1*Δ 3’UTR*_ags1+_::ags1^+^-12A-GFP-12A:leu1^+^:ura4*^+^ *hht1^+^-RFP:KanMX6* h^-^ | This study |
| 4179 | *leu1-32 ura4-*Δ*18 his3-*Δ*1 ade6-M210 sid2-250 hht1^+^-RFP:KanMX6* *ags1*Δ  3’UTR*_ags1+_::ags1^+^-12A-GFP-12A:leu1^+^:ura4*^+^ h^+^ | This study |
| 5469 | *leu1-32 ura4-*Δ*18 his3-*Δ*1 cdc7^+^-GFP:ura4^+^* *hht1^+^-RFP:KanMX6* h^+^ | This study |
| 5473 | *leu1-32 ura4-*Δ*18 cdc25-22* *cdc7^+^-GFP:ura4^+^ hht1^+^-RFP:KanMX6* h^-^ | This study |
| 5705 | *leu1-32 ura4-*Δ*18 csc2*Δ*:KanMX6* *cdc7^+^-GFP:ura4^+^ hht1^+^-RFP:KanMX6* h^-^ | This study |
| 5535 | *leu1-32 ura4-*Δ*18 cps1-191 cdc7^+^-GFP:ura4^+^* *hht1^+^-RFP:KanMX6* h^-^ | This study |
| **Used for experiments in Fig 6, S6 Fig and S7 Fig** | | |
| 2324 | *leu1-32 ura4-*Δ*18* *his3-*Δ*1 ade6-M210 etd1∆::ura4*^+^ P*nmt1^+^*-81X-*etd1^+^:leu1^+^* h^-^ | J. Jiménez^n^ |
| 3599 | *leu1-32 etd1^+^* P*nmt1^+^*-41X-*GFP-etd1^+^:leu1^+^* h^-^ | J. Jiménez^n^ |
| 5452 | *leu1-32 ura4-*Δ*18 etd1^+^* P*nmt1^+^*-41X-*GFP-etd1^+^:leu1^+^ hht1^+^-RFP:KanMX6* h^+^ | This study |
| 5462 | *leu1-32 ura4-*Δ*18 cdc25-22 etd1^+^* P*nmt1^+^*-41X-*GFP-etd1^+^:leu1^+^ hht1^+^-RFP:KanMX6* h^-^ | This study |
| 5659 | *leu1-32 ura4-*Δ*18* *etd1∆::ura4*^+^ P*nmt1^+^*-81X-*etd1^+^:leu1^+^* *hht1^+^-RFP:KanMX6* h^-^ | This study |
| 5853 | *leu1-32 rho1^+^* P*nmt1^+^*-3X-*rho1^+^:leu1^+^* h^-^ | P. Pérez |
| 5877 | *leu1-32 his3-*Δ*1 rho1^+^* P*nmt1^+^*-3X-*rho1^+^:leu1^+^* *hht1^+^-RFP:KanMX6* h^-^ | This study |
| 6131 | *leu1-32 ura4-*Δ*18 etd1^+^* P*nmt1^+^*-41X-*GFP-etd1^+^:leu1^+^ rlc1^+^-tdTom:KanMX6 hht1^+^-RFP:KanMX6* h^-^ | This study |
| 6138 | *leu1-32 ura4-*Δ*18 etd1∆::ura4*^+^ P*nmt1^+^*-41X-*GFP*-*etd1*Δ*1-400:leu1^+^ hht1^+^-RFP:KanMX6* h^+^ | This study |
| ^a^ Institute of General Microbiology, University of Bern, Switzerland.  ^b^ The Francis Crick Institute, Lincoln's Inn Fields Laboratories, London UK.  ^c^ School of Life Sciences, École Polytechnique Fédérale de Lausanne (EPFL), Lausanne, Switzerland.  ^d^ Division of Biomedical Sciences, Warwick Medical School, University of Warwick, Coventry, UK.  ^e^ Division of Biology, School of Arts and Sciences, University of Tokyo, Tokyo, Japan.  ^f^ Department of Cell & Tissue Biology, University of California, San Francisco, CA, USA.  ^g^ Laboratory of Biochemistry and Molecular Biology, National Cancer Institute, NIH, Bethesda, MD, USA.  ^h^ Department of Molecular, Cellular and Developmental Biology, Yale University, New Haven, CT, USA.  ^i^ Institute of Molecular Biosciences, Massey University, Palmerston North, New Zealand.  ^j^ Instituto de Biología Funcional y Genómica, CSIC/Universidad de Salamanca, Salamanca, Spain.  ^k^ Department of Molecular Biotechnology, Hiroshima Research Center for Healthy Aging (HiHA), Graduate School of Advanced Sciences of Matter, Hiroshima University, Higashi-Hiroshima, Japan.  ^l^ Department of Biochemistry and Molecular Pharmacology, University of Massachusetts Medical School, Worcester, MA, USA.  ^m^ Okinawa Institute of Science and Technology Graduate University, Okinawa, Japan.  ^n^ Centro Andaluz de Biología del Desarrollo. CSIC/Universidad Pablo de Olavide, Sevilla, Spain. | | |
